# Supplementary material for: Diagnostic testing intensity for Legionnaires’ disease: Spatio-temporal assessment and its effect on surveillance case reporting, Denmark, 2014–2022
Source: PLoS One. 2026 Apr 17;21(4):e0346580. doi: 10.1371/journal.pone.0346580 (PMC13089883; doi:10.1371/journal.pone.0346580)
Supplement: S1 Appendix — (PDF) [file pone.0346580.s001.pdf]

S1 Appendix - Diagnostic testing intensity for Legionnaires' disease: spatio-temporal assessment and its effect on surveillance case reporting, Denmark, 2014-2022

Emmanuel Robesyn*, Søren Anker Uldum, Karsten Dalsgaard Bjerre, Charlotte Kjelsø, Marc Struelens, Cecilia Stålsby Lundborg, Steen Ethelberg, Christel Faes
* emmanuel.robesyn@ki.se

Table of Contents

S1 Directed Acyclic Graph (DAG)

S2 Reverse seasonality testing versus case reporting

S3 Provinces: map

S4 Municipalities: map, neighborhood graph, and population size

S5 Population: age groups, sex, province, by year

S6 Test profile of tested persons, by year and by province

S7 Tested persons and reported cases, national and by province

S8 Model specification spatial effects

S9 Model comparison testing intensity

S10 Model comparison case reporting, adjusted for testing intensity

S11 Testing - relative testing intensity

S12 Testing - spatial effects

S13 Testing - spatio-temporal interaction effect

S14 Effect of testing intensity on case reporting - alternative binning testing

S15 Reporting, testing-adjusted - relative case reporting

S16 Reporting, testing-adjusted - spatial effects (correlated and independent)

S17 Reporting, testing-adjusted - spatio-temporal interaction effect

S18 Reporting, testing-unadjusted - relative case reporting

S19 Reporting, testing-unadjusted - (absolute) case reporting

S20 Sensitivity analysis

S21 Tables: testing intensity

S22 Tables: testing-adjusted case reporting

S23 Tables: testing-unadjusted case reporting

S24 Tables: difference, testing-adjusted minus testing-unadjusted case reporting

S1 Directed Acyclic Graph (DAG)

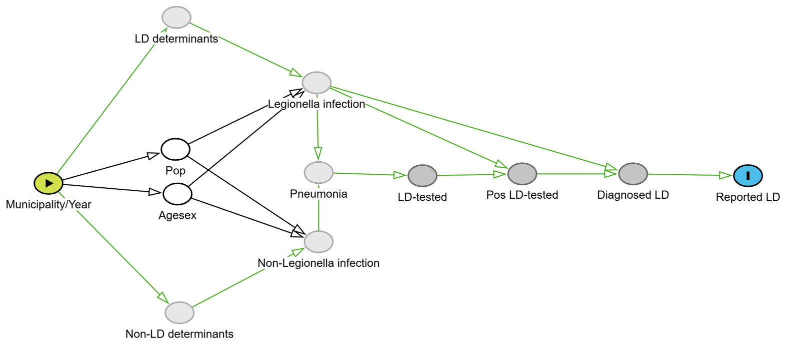

Figure 1. DAG - total effect, no adjustment testing

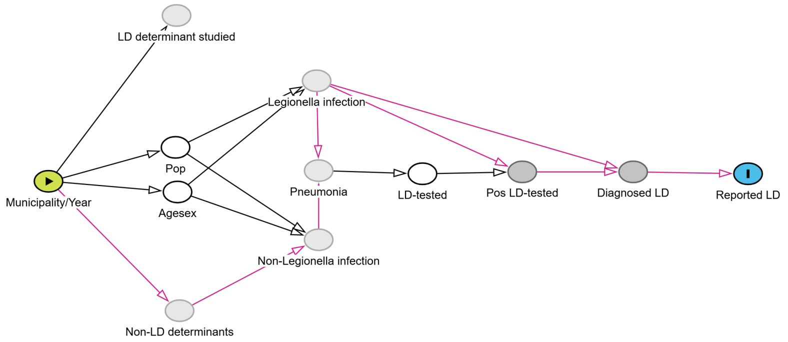

Figure 2. DAG - bias under null from adjustment testing

On the Figure 2 the arrow from the ficticious Legionnaires' disease determinant under study to true Legionnaires' disease was removed to illustrate that bias is present under the null, i.e. an association between exposure and outcome even if there is no effect of the determinant under study.

This bias would not be present if the total volume of pneumonia was independent

(i.e. no arrow) of the Legionnaires' disease occurrence. This is not the case, however only about 4.6% of CAP was estimated to be due to *Legionella*. Sensitivity studies may shed light on the impact of this proportion on the size of bias.

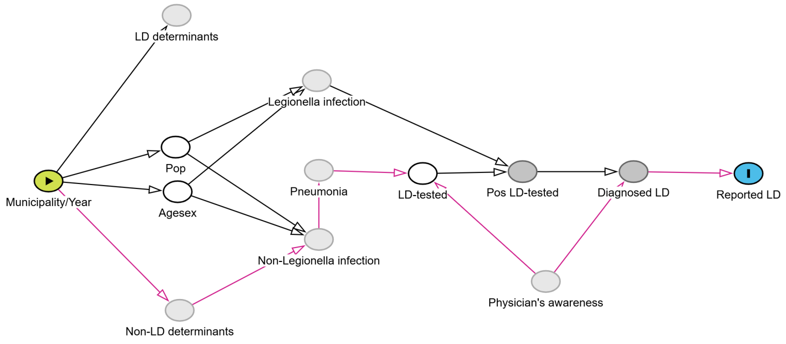

Figure 3. DAG - bias under null from unmeasured confounder

On Figure 3, we also removed the arrow from (the count of) *Legionella* infections to the (total count of) pneumonia, to remove the bias from the previous picture, and to illustrate better the bias from the unmeasured confounder (e.g. awareness due to detection and reporting of cases). Physician's awareness is expected to cause time varying confounding; an aspect not addressed in this study.

S2 Seasonality testing 41

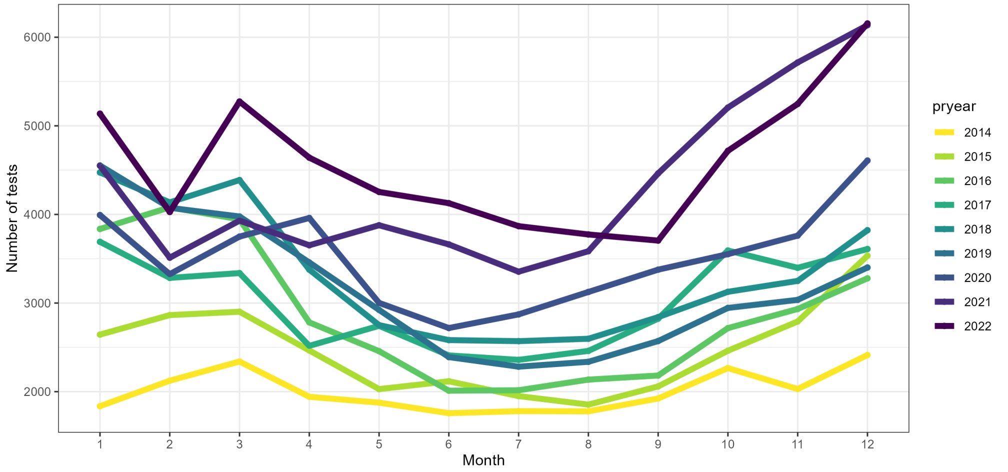

Figure 4. Seasonality of Legionnaires' disease testing, Denmark 2014-2022, opposite of disease occurrence

S3 Provinces: map 42

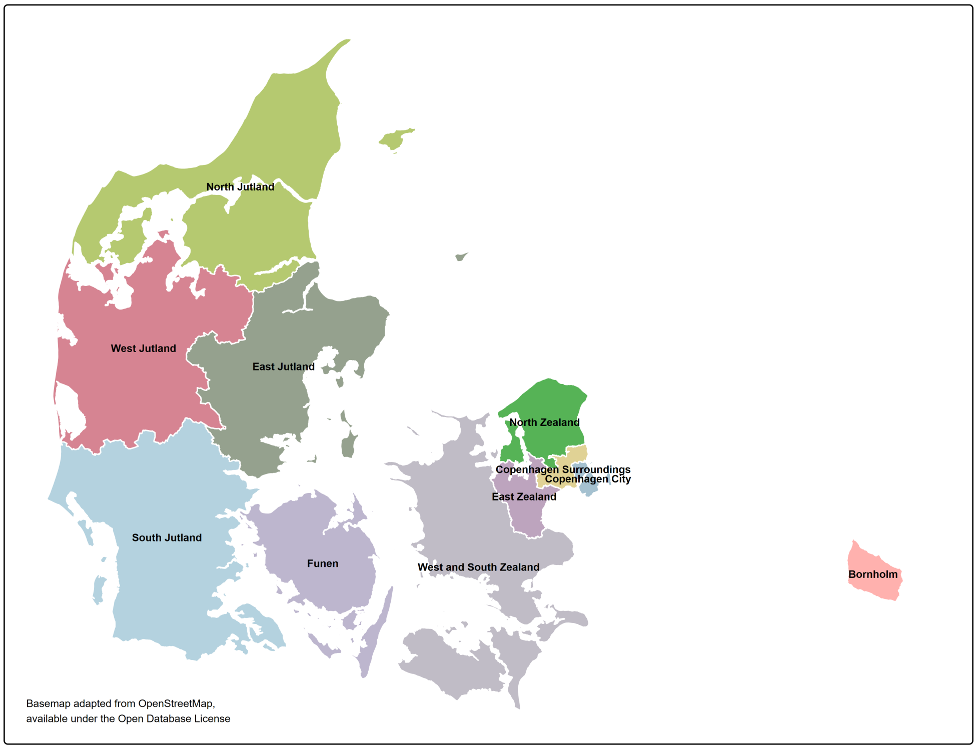

Figure 5. Provinces (11)

S4 Municipalities: map, neighbourhood graph, and population size

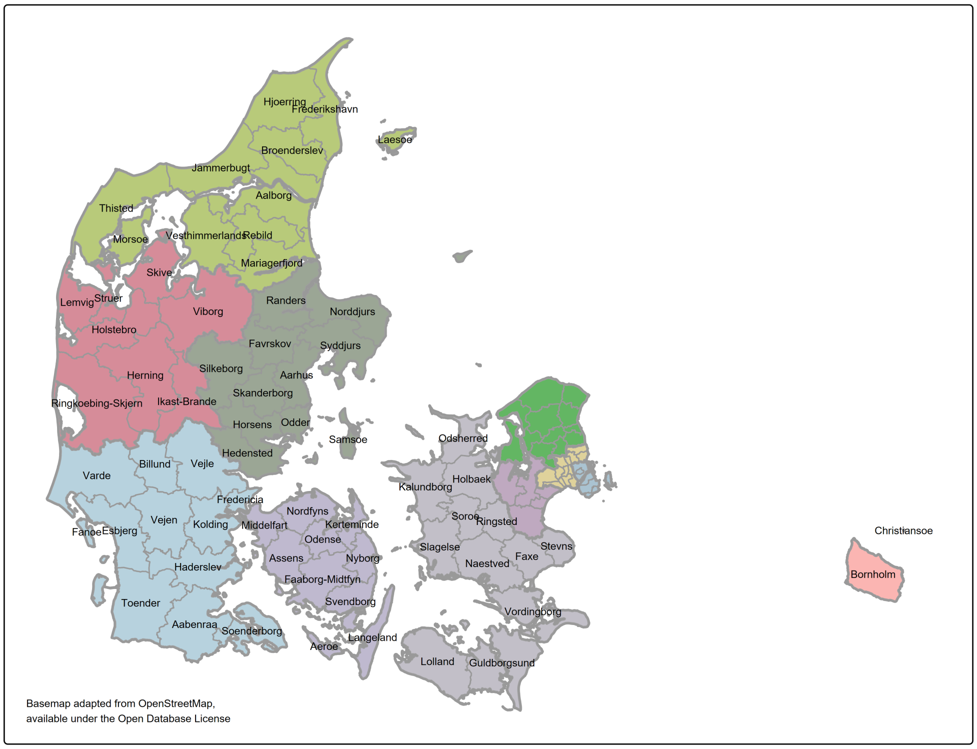

Figure 6. Municipalities (99)

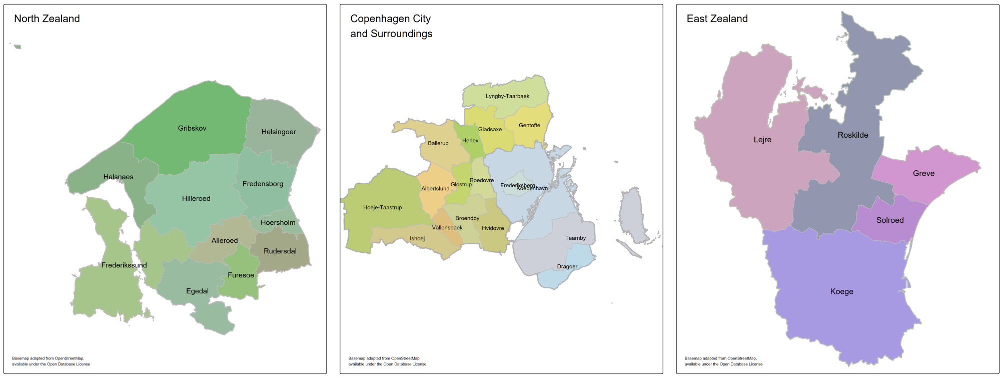

Figure 7. Inset, municipalities of four provinces

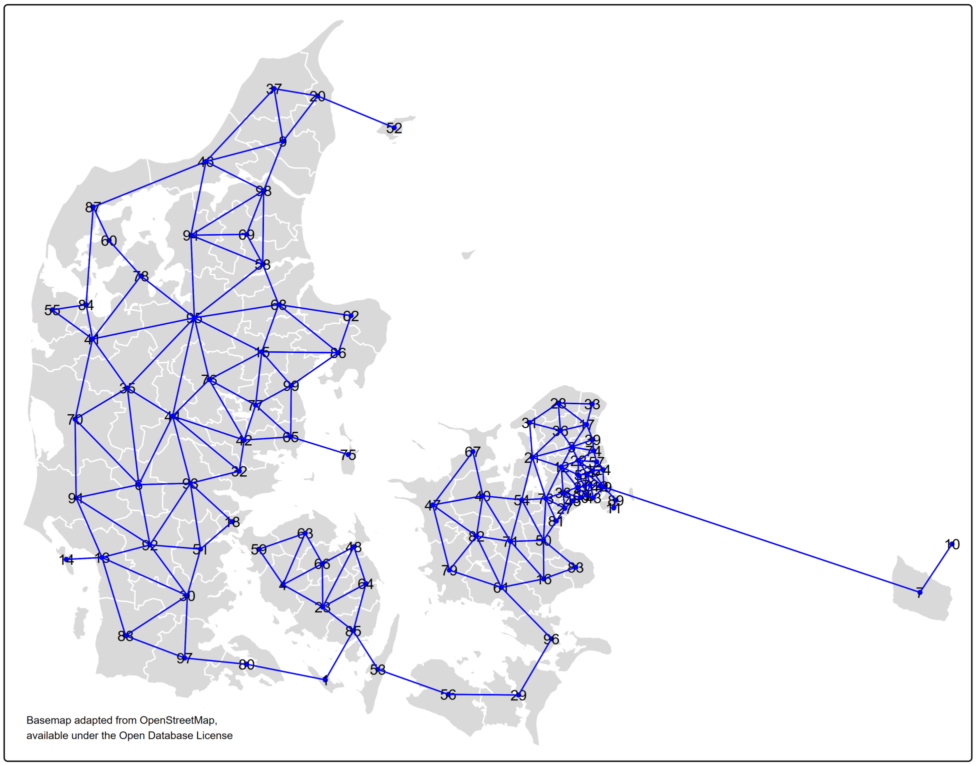

Figure 8. Neighbourhood graph for spatial model

Table 1. Municipalities, by province, and population size (2021)

Province	ID	Municipality	Population (2021)
Bornholm	7	Bornholm	39570
Bornholm	10	Christiansoe	90
Funen	1	Aeroe	5960
Funen	4	Assens	40867
Funen	23	Faaborg-MidtFyn	51683
Funen	48	Kerteminde	23847
Funen	53	Langeland	12367
Funen	59	Middelfart	39116
Funen	63	Nordfyns	29549
Funen	64	Nyborg	31933
Funen	66	Odense	205509
Funen	85	Svendborg	58588
Copenhagen City	11	Dragoer	14569
Copenhagen City	19	Frederiksberg	103677
Copenhagen City	49	Koebenhavn	638117
Copenhagen City	89	Taarnby	42670
Copenhagen Surroundings	2	Albertslund	27366
Copenhagen Surroundings	5	Ballerup	49310
Copenhagen Surroundings	8	Broendby	35232
Copenhagen Surroundings	24	Gentofte	74550
Copenhagen Surroundings	25	Gladsaxe	69200

Province	ID	Municipality	Population (2021)
Copenhagen Surroundings	26	Glostrup	23380
Copenhagen Surroundings	34	Herlev	28913
Copenhagen Surroundings	38	Hoeje-Taastrup	51729
Copenhagen Surroundings	43	Hvidovre	53451
Copenhagen Surroundings	45	Ishoej	23131
Copenhagen Surroundings	57	Lyngby-Taarbaek	56614
Copenhagen Surroundings	72	Roedovre	41113
Copenhagen Surroundings	90	Vallensbaek	16515
North Jutland	9	Broenderslev	36177
North Jutland	20	Frederikshavn	59039
North Jutland	37	Hjoerring	64155
North Jutland	46	Jammerbugt	38175
North Jutland	52	Laesoe	1764
North Jutland	58	Mariagerfjord	41536
North Jutland	60	Morsoe	20066
North Jutland	69	Rebild	30518
North Jutland	87	Thisted	43160
North Jutland	94	Vesthimmerlands	36362
North Jutland	98	Aalborg	219487
North Zealand	3	Alleroed	25893
North Zealand	12	Egedal	43696
North Zealand	17	Fredensborg	40998
North Zealand	21	Frederikssund	45439
North Zealand	22	Furesoe	41001
North Zealand	28	Gribskov	40971
North Zealand	31	Halsnaes	31420
North Zealand	33	Helsingoer	63000
North Zealand	36	Hilleroed	51528
North Zealand	39	Hoersholm	24917
North Zealand	74	Rudersdal	57024
East Jutland	15	Favrskov	48381
East Jutland	32	Hedensted	46773
East Jutland	42	Horsens	92229
East Jutland	62	Norddjurs	36943
East Jutland	65	Odder	22979
East Jutland	68	Randers	98190
East Jutland	75	Samsoe	3682
East Jutland	76	Silkeborg	95488
East Jutland	77	Skanderborg	63390
East Jutland	86	Syddjurs	43168
East Jutland	99	Aarhus	352751
East Zealand	27	Greve	50514
East Zealand	50	Koege	61475
East Zealand	54	Lejre	28173
East Zealand	73	Roskilde	88889
East Zealand	81	Solroed	23441
South Jutland	6	Billund	26551
South Jutland	13	Esbjerg	115579
South Jutland	14	Fanoe	3456
South Jutland	18	Fredericia	51275
South Jutland	30	Haderslev	55376

Province	ID	Municipality	Population (2021)
South Jutland	51	Kolding	93161
South Jutland	80	Soenderborg	73831
South Jutland	88	Toender	37050
South Jutland	91	Varde	49628
South Jutland	92	Vejen	42790
South Jutland	93	Vejle	116992
South Jutland	97	Aabenraa	58526
West and South Zealand	16	Faxe	36713
West and South Zealand	29	Guldborgsund	60328
West and South Zealand	40	Holbaek	71913
West and South Zealand	47	Kalundborg	48487
West and South Zealand	56	Lolland	40539
West and South Zealand	61	Naestved	83181
West and South Zealand	67	Odsherred	32923
West and South Zealand	71	Ringsted	34847
West and South Zealand	79	Slagelse	79122
West and South Zealand	82	Soroe	29993
West and South Zealand	83	Stevns	23034
West and South Zealand	96	Vordingborg	45268
West Jutland	35	Herning	89238
West Jutland	41	Holstebro	58662
West Jutland	44	Ikast-Brande	41473
West Jutland	55	Lemvig	19607
West Jutland	70	Ringkoebing-Skjern	56182
West Jutland	78	Skive	45425
West Jutland	84	Struer	20808
West Jutland	95	Viborg	96679

S5 Population: age groups, sex, province, by year

Table 2. Population description

	2014 (N=5627235)	2015 (N=5659715)	2016 (N=5707251)	2017 (N=5748769)	2018 (N=5781190)	2019 (N=5806081)	2020 (N=5822763)	2021 (N=5840045)	2022 (N=5873420)
**Age group**									
00-49	3524977 (62.6%)	3520891 (62.2%)	3530722 (61.9%)	3532038 (61.4%)	3530908 (61.1%)	3529573 (60.8%)	3521957 (60.5%)	3514493 (60.2%)	3521097 (59.9%)
50-59	737542 (13.1%)	750878 (13.3%)	765248 (13.4%)	781703 (13.6%)	792624 (13.7%)	797775 (13.7%)	800444 (13.7%)	801166 (13.7%)	805849 (13.7%)
60-69	694294 (12.3%)	688697 (12.2%)	681207 (11.9%)	672152 (11.7%)	665223 (11.5%)	662929 (11.4%)	663646 (11.4%)	667583 (11.4%)	673950 (11.5%)
70-79	435531 (7.7%)	459840 (8.1%)	486316 (8.5%)	513155 (8.9%)	535741 (9.3%)	552058 (9.5%)	564390 (9.7%)	574697 (9.8%)	581036 (9.9%)
over 80 y	234891 (4.2%)	239409 (4.2%)	243758 (4.3%)	249721 (4.3%)	256694 (4.4%)	263746 (4.5%)	272326 (4.7%)	282106 (4.8%)	291488 (5.0%)
**Sex**									
female	2834956 (50.4%)	2848701 (50.3%)	2869364 (50.3%)	2888591 (50.2%)	2904717 (50.2%)	2917008 (50.2%)	2925845 (50.2%)	2935188 (50.3%)	2950505 (50.2%)
male	2792279 (49.6%)	2811014 (49.7%)	2837887 (49.7%)	2860178 (49.8%)	2876473 (49.8%)	2889073 (49.8%)	2896918 (49.8%)	2904857 (49.7%)	2922915 (49.8%)
**Province of residence**									
Bornholm	40305 (0.7%)	39919 (0.7%)	39847 (0.7%)	39773 (0.7%)	39715 (0.7%)	39662 (0.7%)	39583 (0.7%)	39660 (0.7%)	39638 (0.7%)
Copenhagen City	728243 (12.9%)	739977 (13.1%)	752964 (13.2%)	764816 (13.3%)	775033 (13.4%)	784618 (13.5%)	794128 (13.6%)	799033 (13.7%)	805402 (13.7%)
Copenhagen Surroundings	530612 (9.4%)	535355 (9.5%)	539241 (9.4%)	542601 (9.4%)	546059 (9.4%)	547534 (9.4%)	548370 (9.4%)	550504 (9.4%)	553622 (9.4%)
East Jutland	851769 (15.1%)	857030 (15.1%)	865830 (15.2%)	875084 (15.2%)	883364 (15.3%)	890567 (15.3%)	897129 (15.4%)	903974 (15.5%)	913861 (15.6%)
East Zealand	239016 (4.2%)	241870 (4.3%)	244698 (4.3%)	246594 (4.3%)	247774 (4.3%)	249359 (4.3%)	250702 (4.3%)	252492 (4.3%)	254400 (4.3%)
Funen	486709 (8.6%)	488578 (8.6%)	491474 (8.6%)	494049 (8.6%)	496243 (8.6%)	498481 (8.6%)	498506 (8.6%)	499419 (8.6%)	501782 (8.5%)
North Jutland	581057 (10.3%)	582632 (10.3%)	585499 (10.3%)	587335 (10.2%)	589148 (10.2%)	589755 (10.2%)	589936 (10.1%)	590439 (10.1%)	591740 (10.1%)
North Zealand	450245 (8.0%)	452874 (8.0%)	457122 (8.0%)	460214 (8.0%)	461852 (8.0%)	463748 (8.0%)	463942 (8.0%)	465887 (8.0%)	469286 (8.0%)
South Jutland	715800 (12.7%)	717150 (12.7%)	720296 (12.6%)	723175 (12.6%)	724520 (12.5%)	724867 (12.5%)	724599 (12.4%)	724215 (12.4%)	726580 (12.4%)
West and South Zealand	577710 (10.3%)	578610 (10.2%)	582801 (10.2%)	585959 (10.2%)	587250 (10.2%)	587379 (10.1%)	586657 (10.1%)	586348 (10.0%)	589113 (10.0%)
West Jutland	425769 (7.6%)	425720 (7.5%)	427479 (7.5%)	429169 (7.5%)	430232 (7.4%)	430111 (7.4%)	429211 (7.4%)	428074 (7.3%)	427996 (7.3%)

Count (Column Percentage)

S6 Test profile of tested persons: within-year test combination, by year and by province

The below two tables describe the test profile or combination of tests that a tested person has received within a given year (i.e. not by disease episode).

UAT: Urinary Antigen Test, LSP: PCR *Legionella* species, LPN: PCR *Legionella pneumophila*.

Table 3. Within-year test profile of tested persons, by year

	2014 (N=16970)	2015 (N=21541)	2016 (N=25221)	2017 (N=26187)	2018 (N=29359)	2019 (N=28285)	2020 (N=31327)	2021 (N=37955)	2022 (N=40881)
**Test profile**									
nouat_LSP_LPN	264 (1.6%)	325 (1.5%)	339 (1.3%)	360 (1.4%)	423 (1.4%)	514 (1.8%)	599 (1.9%)	1105 (2.9%)	1148 (2.8%)
nouat_LSP_noLpn	2100 (12.4%)	2824 (13.1%)	3201 (12.7%)	3487 (13.3%)	3830 (13.0%)	4014 (14.2%)	3679 (11.7%)	3673 (9.7%)	4818 (11.8%)
nouat_noLsp_LPN	5756 (33.9%)	8484 (39.4%)	11063 (43.9%)	10527 (40.2%)	11963 (40.7%)	14081 (49.8%)	21095 (67.3%)	26798 (70.6%)	27731 (67.8%)
UAT_LSP_LPN	269 (1.6%)	307 (1.4%)	315 (1.2%)	316 (1.2%)	374 (1.3%)	333 (1.2%)	401 (1.3%)	544 (1.4%)	494 (1.2%)
UAT_LSP_noLpn	982 (5.8%)	1227 (5.7%)	1446 (5.7%)	1172 (4.5%)	1382 (4.7%)	1419 (5.0%)	1534 (4.9%)	1893 (5.0%)	2056 (5.0%)
UAT_noLsp_LPN	3019 (17.8%)	3505 (16.3%)	3803 (15.1%)	4235 (16.2%)	4728 (16.1%)	3367 (11.9%)	2019 (6.4%)	2183 (5.8%)	2119 (5.2%)
UAT_noLsp_noLpn	4580 (27.0%)	4869 (22.6%)	5054 (20.0%)	6090 (23.3%)	6659 (22.7%)	4557 (16.1%)	2000 (6.4%)	1759 (4.6%)	2515 (6.2%)

Table 4. Within-year test profile of tested persons, by province

	Bornholm (N=1560)	Copenhagen City (N=39637)	Copenhagen Surroundings (N=32291)	East Jutland (N=26391)	East Zealand (N=8608)	Funen (N=33465)	North Jutland (N=11159)	North Zealand (N=28351)	South Jutland (N=39550)	West and South Zealand (N=20144)	West Jutland (N=16570)
**Test profile**											
nouat_LSP_LPN	29 (1.9%)	2323 (5.9%)	758 (2.3%)	115 (0.4%)	152 (1.8%)	167 (0.5%)	134 (1.2%)	522 (1.8%)	461 (1.2%)	350 (1.7%)	66 (0.4%)
nouat_LSP_noLpn	690 (44.2%)	19989 (50.4%)	6600 (20.4%)	121 (0.5%)	520 (6.0%)	69 (0.2%)	292 (2.6%)	843 (3.0%)	1338 (3.4%)	1063 (5.3%)	101 (0.6%)
nouat_noLsp_LPN	38 (2.4%)	3668 (9.3%)	13530 (41.9%)	12505 (47.4%)	3801 (44.2%)	31076 (92.9%)	6211 (55.7%)	18821 (66.4%)	31624 (80.0%)	7679 (38.1%)	8545 (51.6%)
UAT_LSP_LPN	47 (3.0%)	1283 (3.2%)	558 (1.7%)	63 (0.2%)	131 (1.5%)	72 (0.2%)	102 (0.9%)	381 (1.3%)	236 (0.6%)	341 (1.7%)	139 (0.8%)
UAT_LSP_noLpn	390 (25.0%)	7098 (17.9%)	4468 (13.8%)	35 (0.1%)	112 (1.3%)	9 (0.0%)	43 (0.4%)	156 (0.6%)	399 (1.0%)	330 (1.6%)	71 (0.4%)
UAT_noLsp_LPN	8 (0.5%)	454 (1.1%)	1653 (5.1%)	6906 (26.2%)	1726 (20.1%)	1772 (5.3%)	2151 (19.3%)	5207 (18.4%)	2153 (5.4%)	3832 (19.0%)	3116 (18.8%)
UAT_noLsp_noLpn	358 (22.9%)	4822 (12.2%)	4724 (14.6%)	6646 (25.2%)	2166 (25.2%)	300 (0.9%)	2226 (19.9%)	2421 (8.5%)	3339 (8.4%)	6549 (32.5%)	4532 (27.4%)

S7 Tested persons and reported cases, national and by province

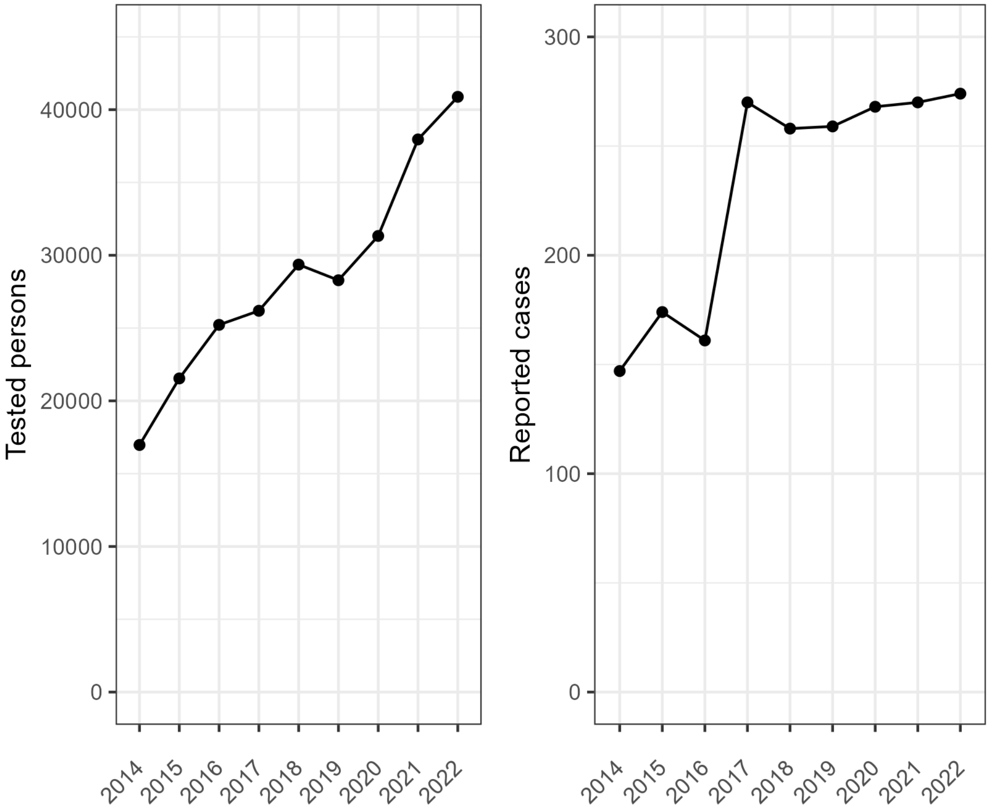

Figure 9. Annual count tested persons (n = 257,729) and reported cases (n = 2,081), national

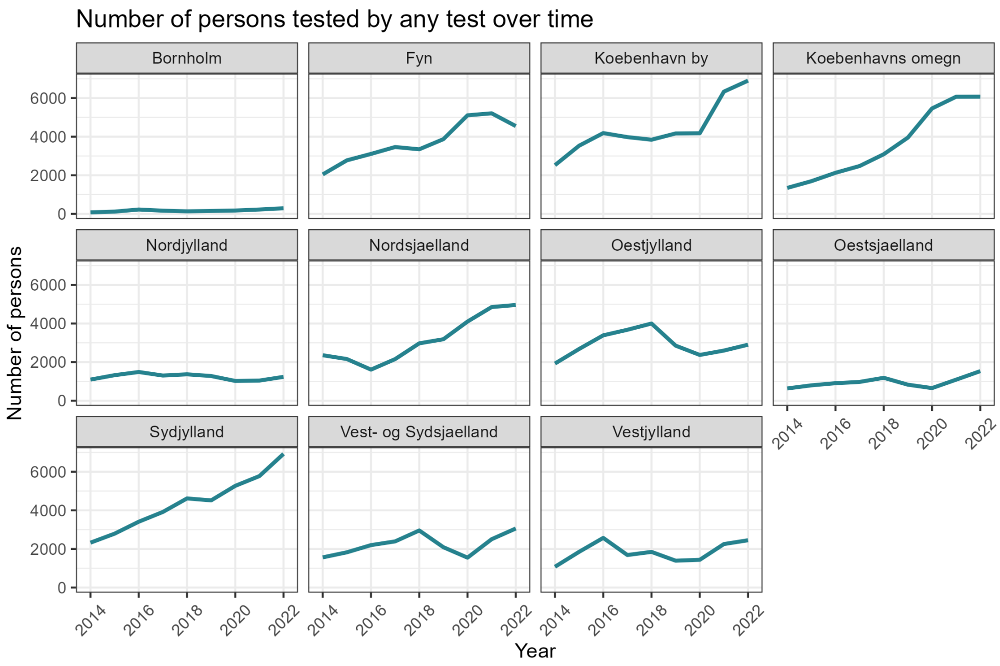

Figure 10. Annual count tested persons, by province

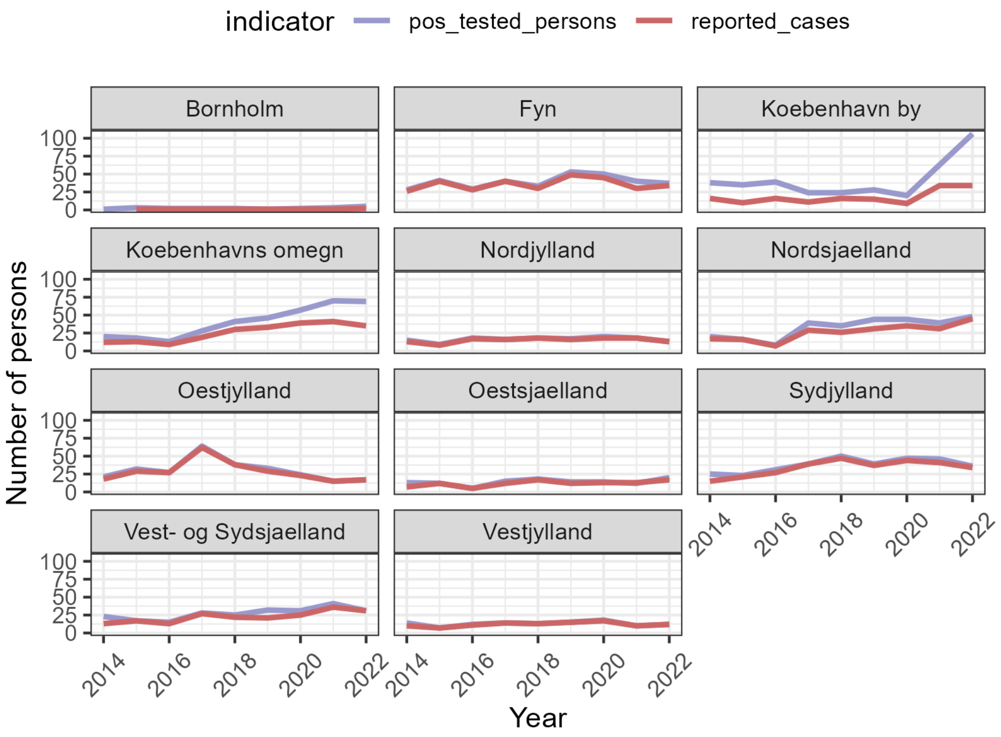

Figure 11. Annual count reported cases, versus positive tested persons, by province

S8 Specification varying effects in model

The spatial varying effect ξ_i is composed of a spatially structured effect u, and a spatially unstructured effect v (Besag, York, Molie), as follows.

$$\xi_i = u_i + v_i$$

$$u_i \sim \text{ICAR}(W, 1/\tau_u) \text{ with W the adjacency matrix of municipalities}$$

$$v_i \sim N(0, 1/\tau_v)$$

where i represents the municipality.

The prior distribution of the temporal varying effect γ_t, and the spatio-temporal interaction effect δ_{it} are as follows.

$$\gamma_t \sim \text{rw}(1)$$

$$\delta_{it} \sim N(0, 1/\tau_\delta)$$

where i represents the municipality and t the year.

Priors for the precision hyperparameters of the varying effects τ were set to Gamma(0.5, 0.0005).

S9 Model comparison testing intensity

Considering our DAG and model equation A of the paper, we compared the fit of a number of functional model variations and provide in the table below the respective WAIC (penalized in-sample fit) and p.eff (overfitting penalty in WAIC). We made models with space as varying effect without time, models with space and time as varying effects, models with space as varying effect and time as fixed effect, and models with space, time and space-time interaction as varying effects. The best model (model A_1), with lowest WAIC, corresponds to a non-linear term for the time component, a combined structured and unstructured spatial effect, and a unstructured space-time interaction.

Table 5. Functional variations of models for testing intensity (equation A in paper)

Model	Formula	WAIC	p.eff
A_26	y ~ 1 + f(s, besag)	174446.398	75348.6737
A_25	y ~ 1 + f(s, bym)	174397.619	75324.1819
A_24	y ~ 1 + f(s, iid)	170504.527	73379.1310
A_23	y ~ 1 + f(t, ar1) + f(s, iid)	110141.543	44793.8569
A_22	y ~ 1 + f(t, rw1) + f(s, iid)	110136.326	44790.8949
A_21	y ~ 1 + f(t, rw1) + f(s, besag)	110125.512	44785.1422
A_20	y ~ 1 + f(t, rw1) + f(s, bym)	110114.200	44779.4814
A_19	y ~ 1 + f(t, ar1) + f(s, bym)	110095.248	44770.4053
A_18	y ~ 1 + f(t, ar1) + f(s, besag)	110067.757	44756.6449
A_17	y ~ 1 + t + f(s, iid)	107406.813	42971.8907
A_16	y ~ 1 + t + f(s, besag)	107083.599	42811.2205
A_15	y ~ 1 + t + f(s, bym)	107020.397	42779.6995
A_14	y ~ 1 + t + f(s, besag) + f(s, t, besag)	35379.712	10758.4499
A_13	y ~ 1 + t + f(s, besag) + f(s, t, bym)	35337.720	10737.7390
A_12	y ~ 1 + t + f(s, bym) + f(s, t, besag)	35328.136	10733.0869
A_11	y ~ 1 + t + f(s, besag) + f(s, t, iid)	35297.341	10718.0108

Model	Formula	WAIC	p.eff
A_10	y ~ 1 + t + f(s, bym) + f(s, t, bym)	35291.269	10714.8207
A_9	y ~ 1 + t + f(s, iid) + f(s, t, besag)	35285.559	10713.0512
A_8	y ~ 1 + t + f(s, bym) + f(s, t, iid)	35278.921	10708.9556
A_7	y ~ 1 + t + f(s, iid) + f(s, t, iid)	35249.966	10695.5038
A_6	y ~ 1 + t + f(s, iid) + f(s, t, bym)	35242.722	10691.5400
A_5	y ~ 1 + f(t, ar1) + f(s, bym) + f(s, t, iid)	31844.135	9309.2344
A_4	y ~ 1 + f(t, ar1) + f(s, iid) + f(s, t, bym)	31835.640	9305.1157
A_3	y ~ 1 + f(t, rw1) + f(s, iid) + f(s, t, bym)	31834.401	9304.4338
A_2	y ~ 1 + f(t, rw1) + f(s, bym) + f(s, t, iid)	31830.661	9302.4233
A_1	y ~ 1 + f(t, rw1) + f(s, bym) + f(st, iid)	7734.934	441.3721

f() random effect, **s** spatial effect, **t** time effect, **st** space-time interaction, **iid** independent identically distributed, **besag** spatial component Besag model , **bym** spatial component Besag-York-Mollie model, **rw** random walk, **ar** autoregressive.

S10 Model comparison case reporting, adjusted for testing intensity

Considering our DAG and model equation B of the paper, we compared the fit of a number of functional model variations and provide in the table below the respective WAIC (penalized in-sample fit) and p.eff (overfitting penalty in WAIC). We made models with space as varying effect without time, models with space and time as varying effects, models with space as varying effect and time as fixed effect, and models with space, time and space-time interaction as varying effects. The best model (model B_1), with lowest WAIC, corresponds to a non-linear term for testing, a non-linear term for the time component, a combined structured and unstructured spatial effect, and a unstructured space-time interaction.

Table 6. Functional variations of models for case reporting (equation B in paper)

Model	Formula	WAIC	p.eff
B_23	y ~ 1 + f(s, iid)	3156.376	82.79889
B_22	y ~ 1 + f(s, besag)	3129.482	61.36332
B_21	y ~ 1 + f(s, bym)	3129.338	61.69408
B_20	y ~ 1 + t + f(s, bym)	3088.689	61.25871
B_19	y ~ 1 + t + f(s, bym) + f(s, t, iid)	3082.675	77.90546
B_18	y ~ 1 + f(t, ar1) + f(s, bym)	3071.044	68.56459
B_17	y ~ 1 + f(t, rw1) + f(s, bym)	3070.488	67.31094
B_16	y ~ 1 + f(t2, rw1) + f(s, bym) + f(s, t, iid)	3061.845	84.76717
B_15	y ~ 1 + t + f(s, bym) + f(s, t, bym)	3047.860	71.59317
B_14	y ~ 1 + t + f(s, bym) + f(s, t, besag)	3044.663	68.31512
B_13	y ~ 1 + f(inla.group(T), rw2) + f(t, rw1) + f(s, bym) + f(st, iid)	3017.084	157.03299
B_12	y ~ 1 + f(t, rw1) + f(s, bym) + f(st, iid)	3000.799	159.30545
B_11	y ~ 1 + T + f(t, rw1) + f(s, bym) + f(st, iid)	2975.420	140.24994
B_10	y ~ 1 + f(inla.group(T), ar1) + f(t, rw1) + f(s, bym) + f(st, iid)	2962.480	129.44414
B_9	y ~ 1 + f(inla.group(T), rw1) + f(t, rw1) + f(s, bym) + f(st, iid)	2961.861	129.28218

Model	Formula	WAIC	p.eff
B_8	y ~ 1 + f(inla.group(T.grp2), ar1) + f(t, rw1) + f(s, bym) + f(st, iid)	2961.350	125.43308
B_7	y ~ 1 + f(inla.group(T.grp2), ar1) + f(t, ar1) + f(s, bym) + f(st, iid)	2960.881	125.56423
B_6	y ~ 1 + f(inla.group(T.grp2), rw1) + f(t, rw1) + f(s, bym) + f(st, iid)	2960.769	125.10365
B_5	y ~ 1 + f(inla.group(T.grp), rw1) + f(t, rw1) + f(s, bym) + f(st, iid)	2960.407	125.17615
B_4	y ~ 1 + f(inla.group(T.grp2), ar1) + f(t, ar1) + f(s, bym) + f(st, iid)	2957.858	122.83940
B_3	y ~ 1 + f(inla.group(T.grp), ar1) + f(t, ar1) + f(s, bym) + f(st, iid)	2957.757	123.38722
B_2	y ~ 1 + f(inla.group(T.grp2), rw1) + f(t, ar1) + f(s, bym) + f(st, iid)	2957.238	122.90229
B_1	y ~ 1 + f(inla.group(T.grp), rw1) + f(t, ar1) + f(s, bym) + f(st, iid)	2956.667	123.11563

f() random effect, **s** spatial effect, **t** time effect, **st** space-time interaction, **T** Testing count, **T.grp** Testing in quantile bins, **T.grp2** Testing in equally spaced bins, **iid** independent identically distributed, **besag** spatial component Besag model , **bym** spatial component Besag-York-Mollie model, **rw** random walk, **ar** autoregressive.

S11 Testing - relative testing intensity

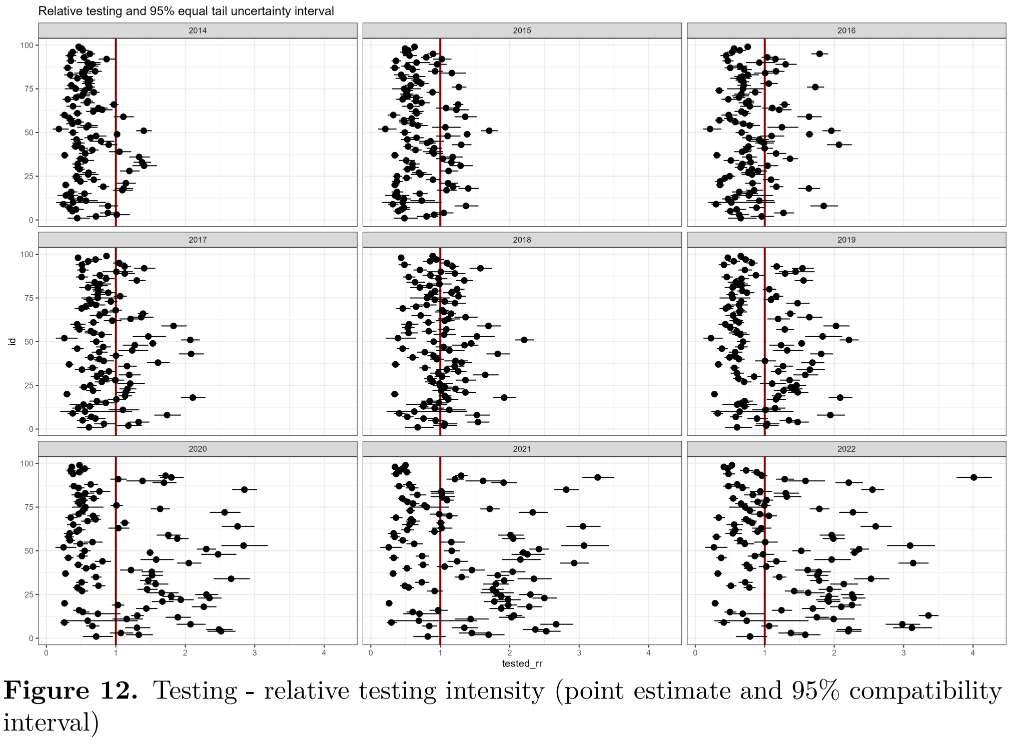

Figure 12. Testing - relative testing intensity (point estimate and 95% compatibility interval)

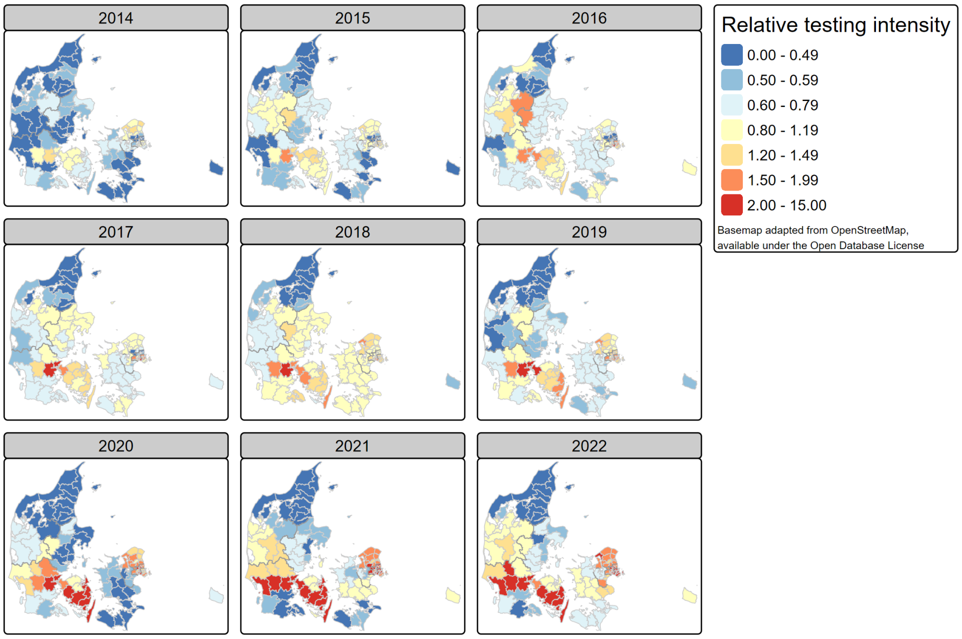

Figure 13. Testing - relative testing intensity (map)

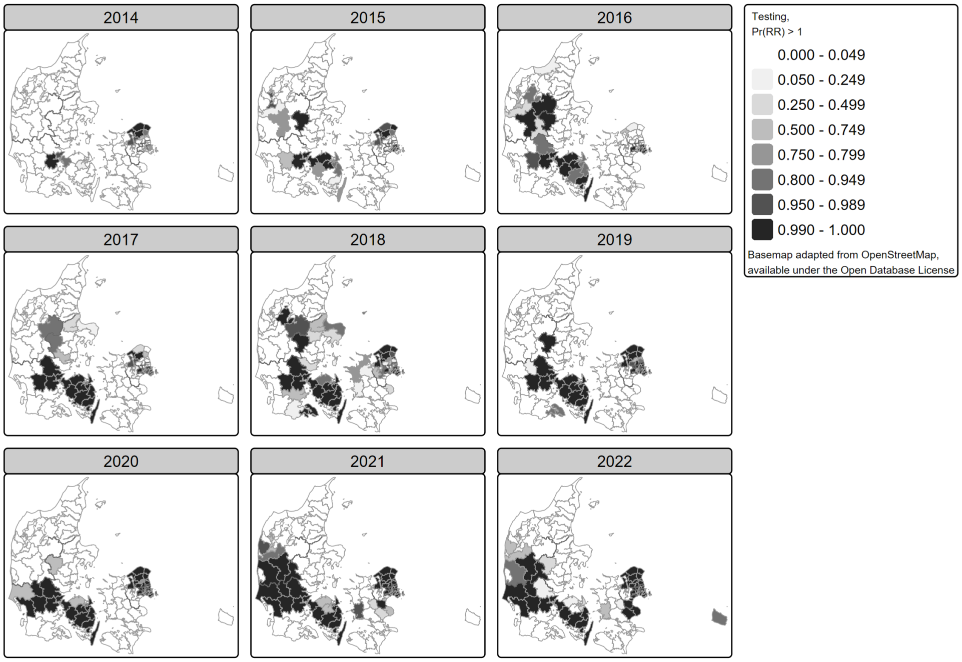

Figure 14. Testing - exceedance probability of relative testing intensity (map). Note: the complement is probability of deficit testing, relative to expected

S12 Testing - spatial effects

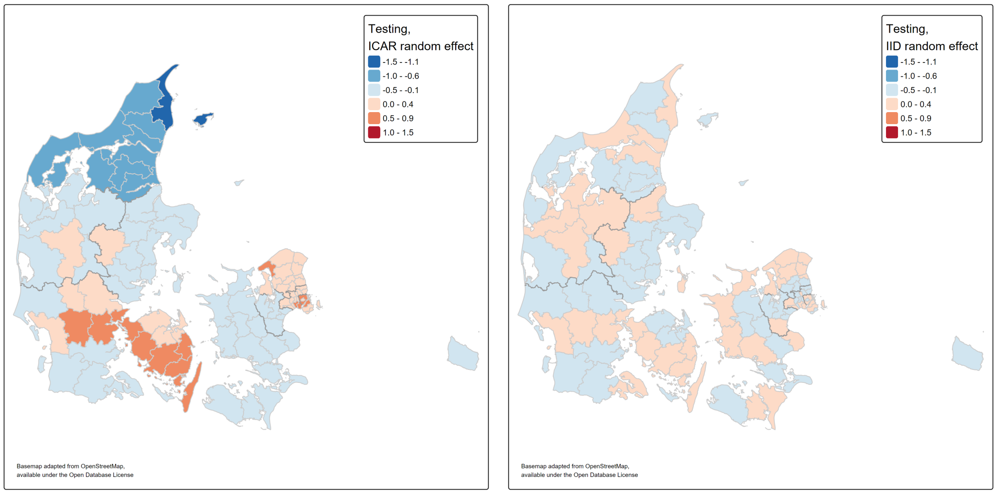

Figure 15. Testing - spatially structured and unstructured effect

S13 Testing - spatio-temporal interaction effect

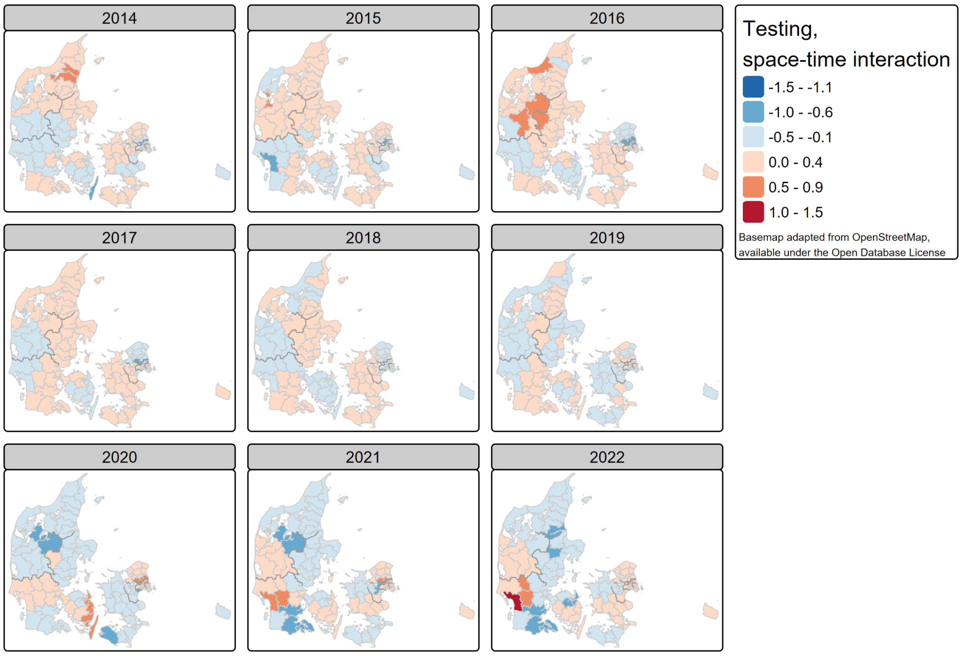

Figure 16. Testing space-time interaction

S14 Effect of testing intensity on case reporting - alternative binning testing intensity

The figure shows the effect of testing intensity on the log scale, according to an alternative testing intensity binning approach than in the paper. Figure 3 (b) in the paper is based on the best model fit (fit 18c) with quantile binning. The below figure is from a nearly equally good model fit (fit 21) with fixed interval binning. This includes extreme testing values up to 2500, however with high uncertainty.

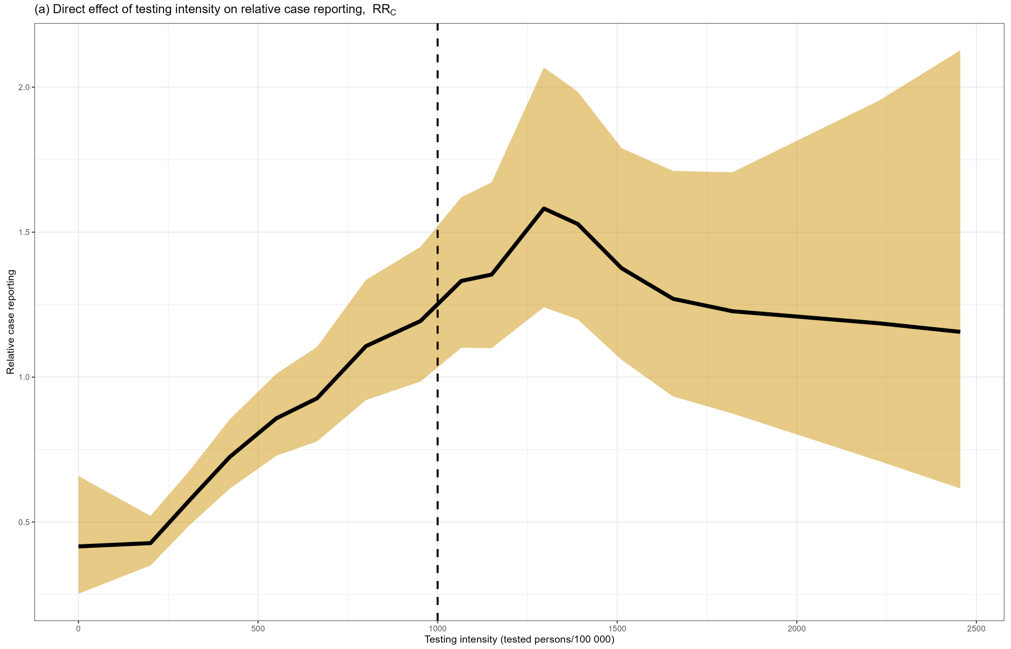

Figure 17. Effect of testing intensity on case reporting

S15 Case reporting, testing-adjusted - relative case reporting

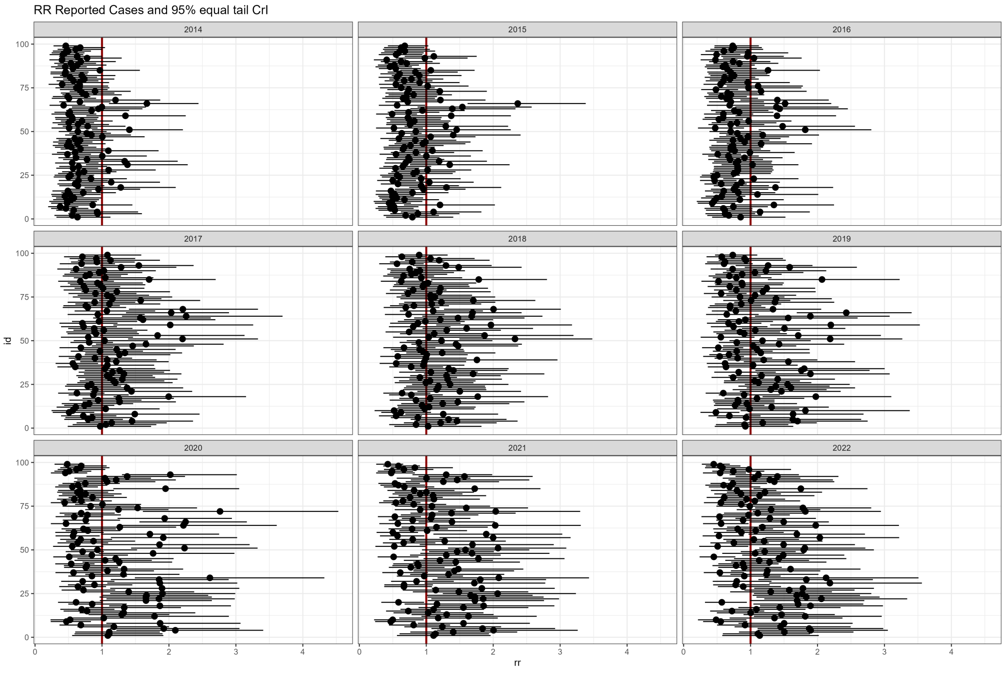

Figure 18. Reporting, testing-adjusted - relative case reporting

S16 Case reporting, testing-adjusted - spatial effects

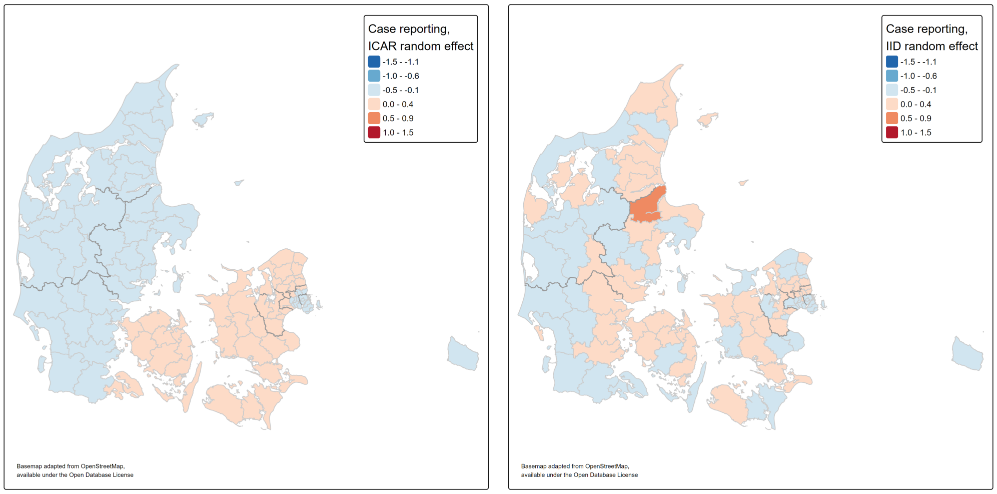

Figure 19. Case reporting spatially structured and unstructured effect

S17 Case reporting, testing-adjusted - spatio-temporal interaction effect

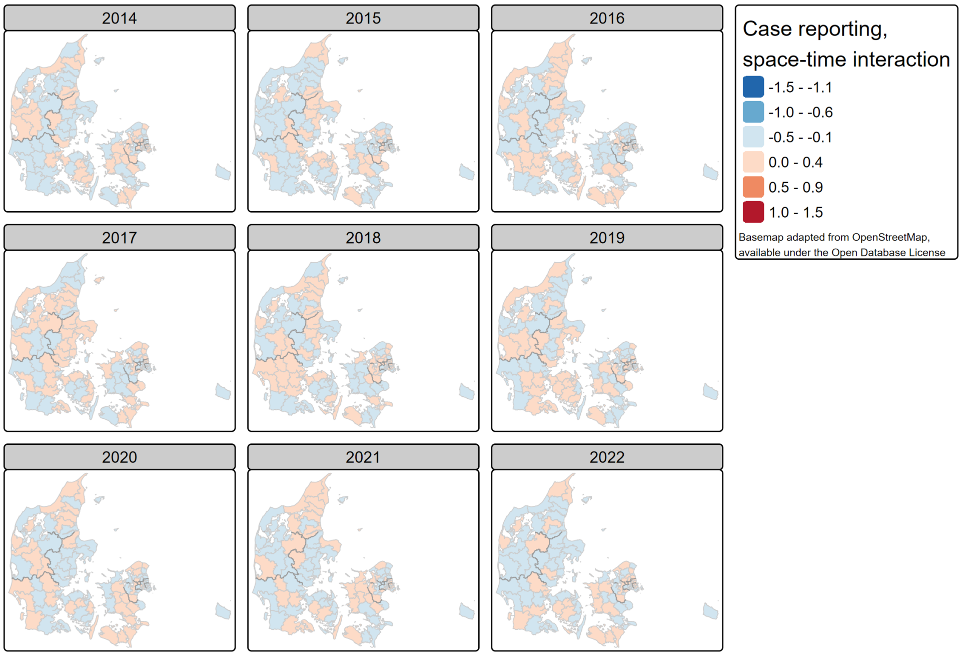

Figure 20. Case reporting space-time interaction

S18 Case reporting, testing-unadjusted - relative case reporting

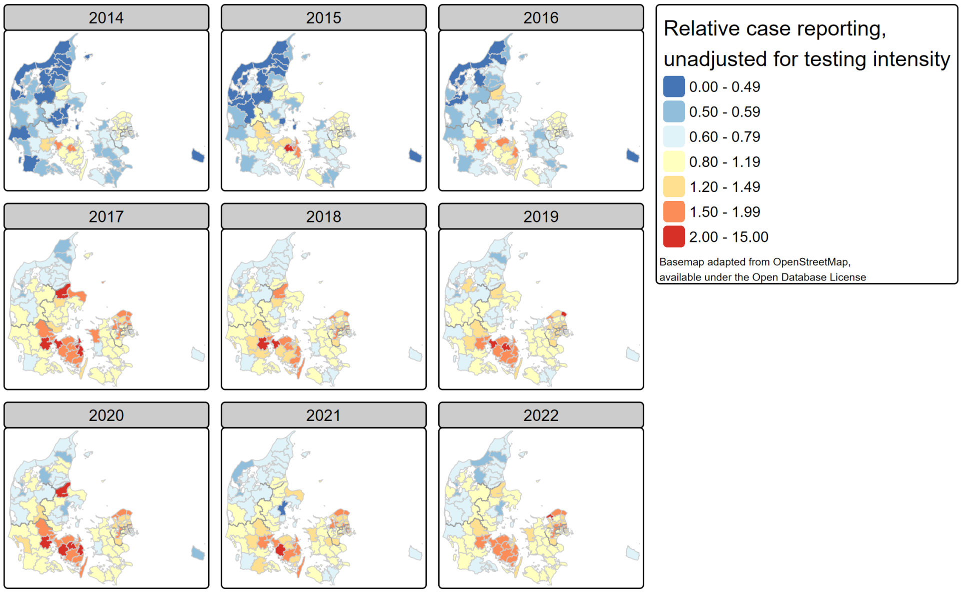

Figure 21. Relative case reporting, not adjusted for testing

S19 Case reporting, testing-unadjusted - case reporting

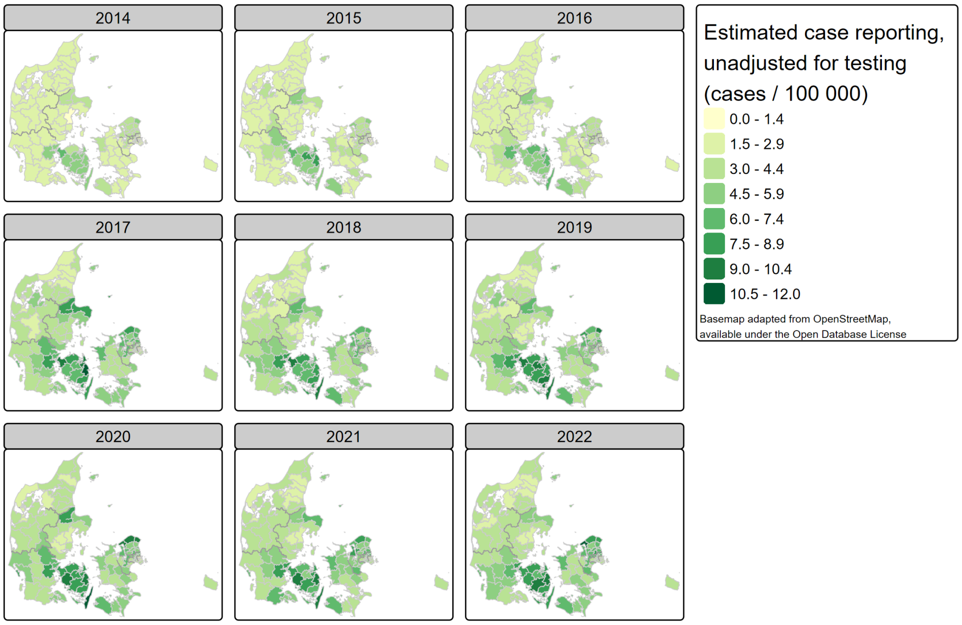

Figure 22. Case reporting, not adjusted for testing

S20 Sensitivity analyses

a) The relative case reporting, in comparison with the relative testing intensity, not adjusted for testing.

When we compare **RR of testing** and **RR of case reporting, not adjusted for testing**, we find notable differences between municipalities (Fig 23).

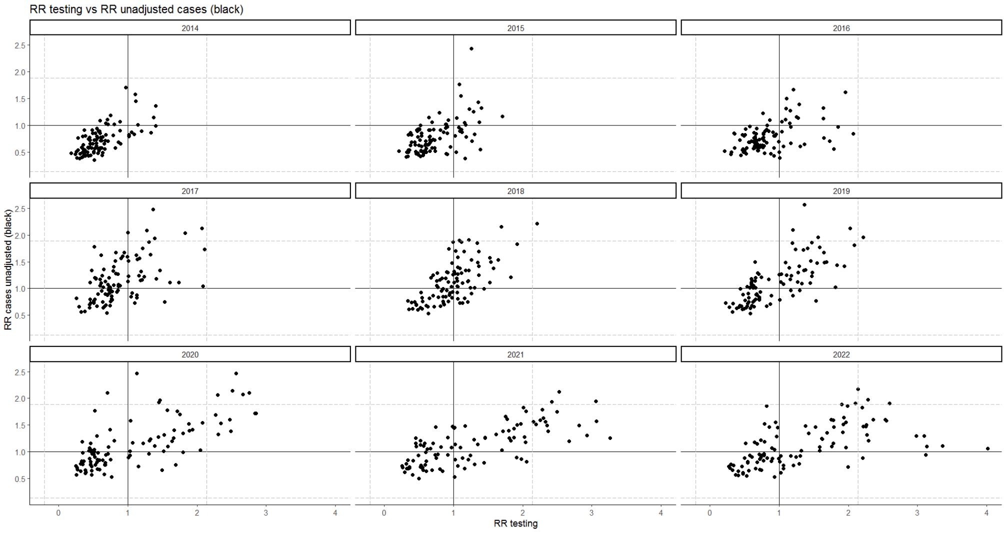

Figure 23. RR testing versus RR testing-unadjusted case reporting of municipalities (black dots)

We can distinguish three municipality profiles of interest, based on the relationships shown in Figure 23.

1. No high relative testing (RR<1.5), and high relative case reporting (RR>2.2)

We found this situation in 2015 in Odense (Funen), in 2017 in Nyborg (Funen), in 2019 in Odense (Funen), and in 2020 in Odense (Funen).

2. High relative testing (RR>2), and high relative case reporting (RR>2.2)

We found this situation in 2018 in Kolding (South Jutland), and in 2020 in Roedovre (Koebenhavns omegn).

3. Very high relative testing (RR>3), and no high relative case reporting (RR<2)

We have found this situation in 2021 in Langeland and Nyborg (Funen) and in Vejen (South Jutland). We have found the same situation in 2022 in Langeland (Funen), Hvidovre (Koebenhavns omegn), and in Vejen, Billund, Esbjerg (South Jutland).

b) The relative case reporting, adjusted versus not adjusted for testing intensity.

When we **also plot the adjusted case reporting (red dots)**, we see that the resulting RR is clearly distinct. Based on the assumptions made, we expect the adjusted RR to be a better estimate of the true latent disease occurrence, however with reservation for other alternative views as expressed in the Discussion section.

When we compare RR of testing and RR of case reporting, we still see an upward trend and this trend does not disappear (entirely) when we adjust the latter for testing.

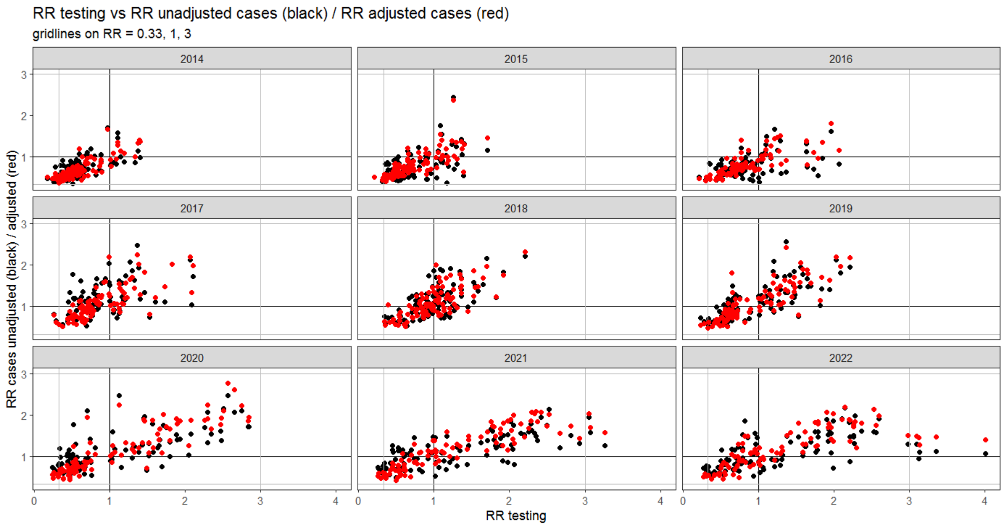

Figure 24. RR testing versus RR testing-unadjusted and -adjusted case reporting

We conclude that areas which have a high relative testing, have generally higher case reporting. Despite the general trend, there are areas where testing is high and still the cases are not so high, e.g. Vejen has a testing RR of 4.01 and the adjusted case RR is 1.4, higher than the unadjusted but still not very high. On the other hand, there are also areas where testing is low and anyway many cases are found. These areas may be of special interest for further study.

c) Sensitivity analysis of case reporting excluding travel-associated Legionnaires' disease cases (i.e. without TALD)

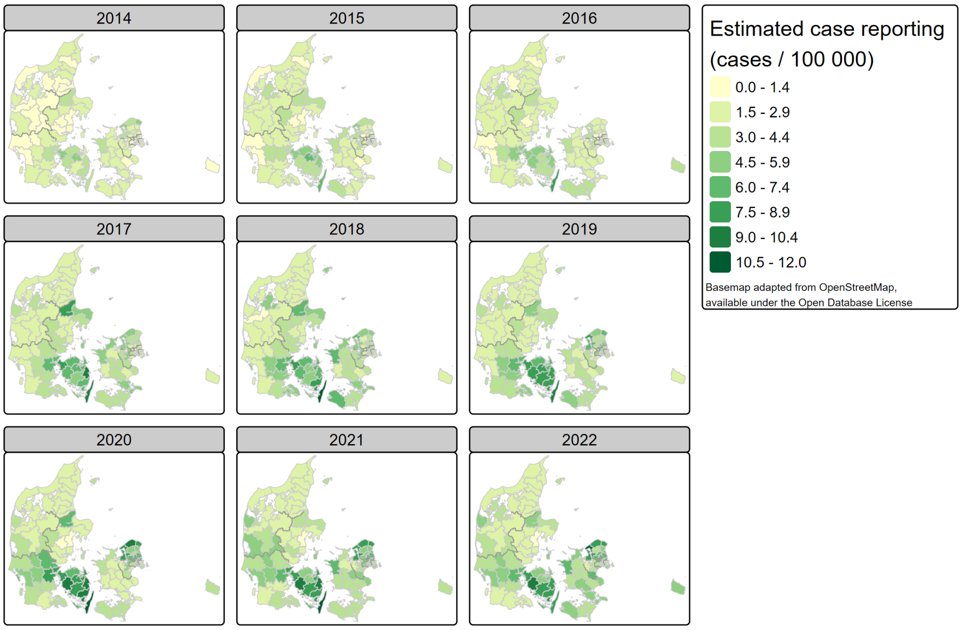

Figure 25. Map of estimated case reporting WITHOUT TALD, adjusted for varying testing intensity, by year and municipality, Denmark 2014-2022.

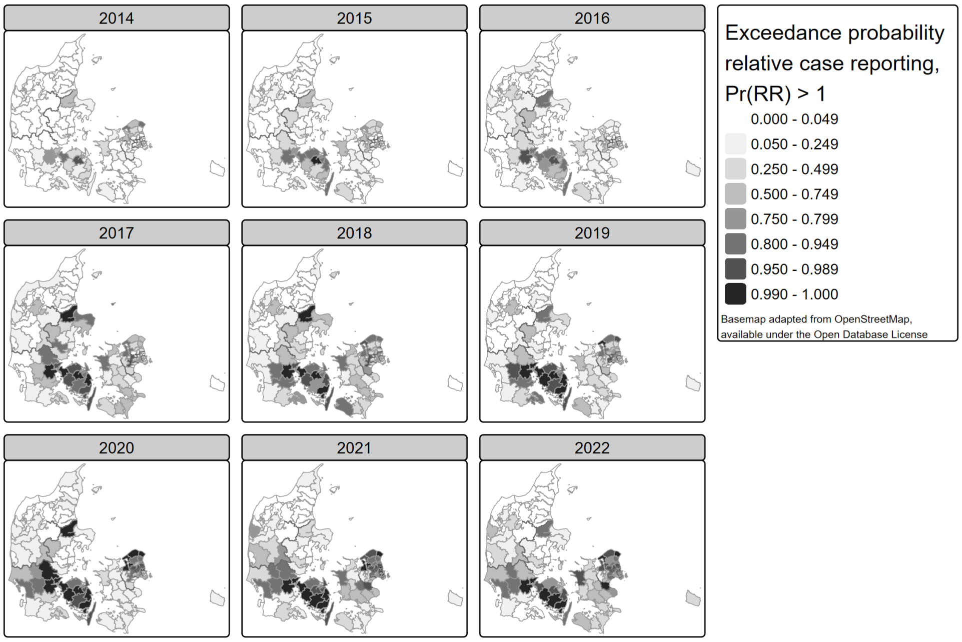

Figure 26. Map of exceedance probability (Pr(RR > 1)) of relative case reporting WITHOUT TALD, adjusted for varying testing intensity, by year and municipality, Denmark 2014-2022.

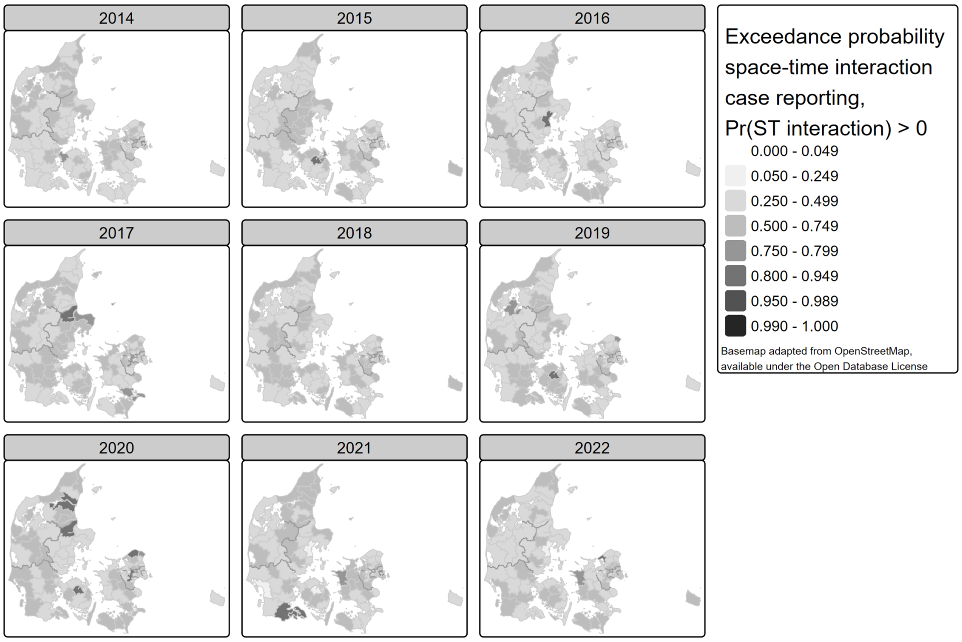

Figure 27. Map of exceedance probability (Pr(STinteraction > 0)) of spatio-temporal interaction of case reporting WITHOUT TALD, adjusted for varying testing intensity, by year and municipality, Denmark 2014-2022.

S21 Testing intensity, look-up table.

Table 7. Testing intensity (estimated): tested persons/100 000 inhabitants.

Province	Municipality	2014	2015	2016	2017	2018	2019	2020	2021	2022
B	Bornholm	202	298	560	417	343	384	443	571	732
B	Christiansoe	198	239	292	302	328	330	334	402	443
F	Aeroe	322	359	490	472	524	475	573	663	642
F	Assens	467	560	687	728	857	830	1442	1486	1307
F	Faaborg-Midtfyn	375	500	615	664	700	819	1394	1503	1389
F	Kerteminde	387	611	617	730	788	737	1506	1401	617
F	Langeland	403	765	897	1070	1127	1368	2160	2398	2446
F	Middelfart	574	714	872	987	932	1132	993	1162	1148
F	Nordfyns	416	646	642	654	612	664	589	594	562
F	Nyborg	408	595	614	767	783	952	1625	1831	1579
F	Odense	427	556	573	625	523	623	520	468	385
F	Svendborg	374	498	635	718	752	884	1651	1649	1508
C.C	Dragoer	313	407	517	623	651	590	677	855	1203
C.C	Frederiksberg	358	520	514	501	476	543	472	921	1062
C.C	Koebenhavn	348	472	558	519	490	521	510	758	798
C.C	Taarnby	324	466	634	552	595	639	843	960	1114

Province	Municipality	2014	2015	2016	2017	2018	2019	2020	2021	2022
C.S	Albertslund	299	344	417	526	480	472	625	820	771
C.S	Ballerup	184	199	258	271	491	715	1333	1273	1200
C.S	Broendby	433	671	913	863	760	980	1059	1367	1521
C.S	Gentofte	216	254	213	270	539	713	943	1000	1045
C.S	Gladsaxe	198	172	226	288	452	667	1067	1070	1056
C.S	Glostrup	293	348	399	593	488	552	826	897	806
C.S	Herlev	271	248	250	253	544	825	1333	1183	1288
C.S	Hoeje-Taastrup	242	396	514	719	592	776	709	961	838
C.S	Hvidovre	414	599	954	966	849	845	963	1384	1499
C.S	Ishoej	315	349	385	511	475	566	689	951	885
C.S	Lyngby-Taarbaek	182	246	178	244	556	660	967	1051	1012
C.S	Roedovre	262	254	330	320	595	720	1253	1140	1114
C.S	Vallensbaek	264	298	425	467	470	679	660	786	787
N-J	Broenderslev	128	167	160	205	224	179	144	168	235
N-J	Frederikshavn	157	200	208	176	208	171	165	169	186
N-J	Hjoerring	138	185	168	181	198	189	159	190	196
N-J	Jammerbugt	228	280	499	243	256	218	183	194	224
N-J	Laesoe	136	155	160	200	315	186	209	222	238
N-J	Mariagerfjord	172	305	262	247	291	282	183	200	180
N-J	Morsoe	149	187	282	264	335	266	208	209	347
N-J	Rebild	147	223	234	252	229	221	178	186	177
N-J	Thisted	161	183	271	278	305	258	228	240	284
N-J	Vesthimmerlands	182	297	241	282	270	236	225	219	291
N-J	Aalborg	230	224	254	209	202	219	172	165	197
N-Z	Alleroed	491	452	317	423	552	541	572	789	760
N-Z	Egedal	211	212	214	210	434	551	927	1009	884
N-Z	Fredensborg	540	552	437	526	592	628	796	1062	971
N-Z	Frederikssund	579	577	435	613	747	825	960	1047	1153
N-Z	Furesoe	249	193	185	351	537	686	1061	1103	1070
N-Z	Gribskov	651	624	514	578	811	826	906	1122	1265
N-Z	Halsnaes	760	711	583	678	957	940	953	1115	1367
N-Z	Helsingoer	716	614	429	493	704	683	857	1144	1086
N-Z	Hilleroed	620	554	368	559	589	625	763	928	896
N-Z	Hoersholm	630	540	417	516	772	645	799	973	1099
N-Z	Rudersdal	304	292	192	348	527	626	952	1003	1058
E-J	Favrskov	247	320	362	401	466	324	257	301	253
E-J	Hedensted	218	293	337	397	500	313	268	393	390
E-J	Horsens	188	245	351	464	423	267	221	367	363
E-J	Norddjurs	368	358	371	537	609	342	282	319	357
E-J	Odder	196	294	414	410	391	301	219	322	329
E-J	Randers	289	316	379	497	521	334	370	308	392
E-J	Samsoe	300	342	499	549	611	408	435	622	559
E-J	Silkeborg	289	600	829	516	621	582	510	395	511
E-J	Skanderborg	158	255	315	337	399	246	227	293	321
E-J	Syddjurs	305	330	392	490	562	390	276	359	420
E-J	Aarhus	184	252	304	350	362	267	196	207	221
E-Z	Greve	288	334	372	396	451	364	270	492	773
E-Z	Koege	178	230	318	348	448	346	283	606	807
E-Z	Lejre	298	339	398	414	561	348	264	344	396
E-Z	Roskilde	319	421	414	449	523	331	266	291	409

Province	Municipality	2014	2015	2016	2017	2018	2019	2020	2021	2022
E-Z	Solroed	204	218	299	286	342	264	256	518	675
S-J	Billund	212	225	304	364	400	472	707	739	1730
S-J	Esbjerg	168	190	282	312	383	366	686	1097	1818
S-J	Fanoe	174	225	507	358	519	419	528	509	513
S-J	Fredericia	543	693	818	1071	991	1093	1206	1242	1154
S-J	Haderslev	233	312	346	391	561	473	426	283	301
S-J	Kolding	647	795	920	986	1068	1085	1146	1222	1209
S-J	Soenderborg	336	360	364	419	714	622	406	275	338
S-J	Toender	348	294	362	440	498	514	384	339	376
S-J	Varde	181	190	248	280	387	396	585	702	756
S-J	Vejen	432	517	591	734	834	823	977	1800	2237
S-J	Vejle	255	379	496	543	573	579	857	656	487
S-J	Aabenraa	272	283	344	386	522	407	315	264	276
WS-Z	Faxe	181	193	329	385	454	386	261	547	710
WS-Z	Guldborgsund	277	311	485	513	517	382	281	348	504
WS-Z	Holbaek	319	316	359	384	485	326	302	280	426
WS-Z	Kalundborg	337	353	419	454	590	377	298	439	518
WS-Z	Lolland	283	301	365	412	545	395	230	316	450
WS-Z	Naestved	219	330	359	346	430	329	235	499	496
WS-Z	Odsherred	358	453	461	538	688	425	403	417	546
WS-Z	Ringsted	232	246	300	335	382	302	226	490	467
WS-Z	Slagelse	299	349	356	390	484	361	286	602	570
WS-Z	Soroe	275	330	347	386	446	355	244	369	464
WS-Z	Stevns	186	240	421	437	570	364	334	620	797
WS-Z	Vordingborg	214	286	312	357	512	342	220	268	479
W-J	Herning	227	486	647	320	380	280	361	674	632
W-J	Holstebro	245	446	495	320	306	254	351	562	548
W-J	Ikast-Brande	216	390	485	378	391	294	418	654	620
W-J	Lemvig	207	341	399	400	326	370	417	746	662
W-J	Ringkoebing-Skjern	219	342	332	302	354	262	379	649	616
W-J	Skive	314	387	574	493	654	427	272	317	548
W-J	Struer	275	634	557	394	367	346	465	637	597
W-J	Viborg	298	433	874	518	549	415	247	276	477

Provinces: **B** Bornholm, **F** Funen, **C.C** Copenhagen City, **C.S** Copenhagen Surroundings, **N-J** North Jutland, **N-Z** North Zealand, **E-J** East Jutland, **E-Z** East Zealand, **S-J** South Jutland, **WS-Z** West and South Zealand, **W-J** West Jutland. **S-J** South Jutland, **WS-Z** West and South Zealand, **W-J** West Jutland.

S22 Testing-adjusted case reporting, look-up table.

Table 8. Case reporting (estimated): reported cases/100 000 inhabitants.

Province	Municipality	2014	2015	2016	2017	2018	2019	2020	2021	2022
B	Bornholm	1.93	2.73	3.98	3.92	3.02	3.80	3.85	4.62	5.29
B	Christiansoe	2.18	2.24	2.26	2.78	2.45	8.34	2.63	2.50	2.48
F	Aeroe	3.88	4.96	5.39	6.34	6.77	6.15	7.31	7.55	7.80
F	Assens	4.02	4.88	5.10	6.58	6.71	7.93	9.96	9.77	9.34

Province	Municipality	2014	2015	2016	2017	2018	2019	2020	2021	2022
F	Faaborg-Midtfyn	3.78	4.25	4.88	6.46	5.57	7.71	8.13	8.56	9.19
F	Kerteminde	3.76	6.40	5.48	7.84	6.98	7.03	8.88	8.65	5.57
F	Langeland	4.65	7.79	9.01	11.29	11.68	10.80	11.95	11.13	9.96
F	Middelfart	5.76	5.95	6.14	9.01	8.95	10.17	8.03	9.06	8.17
F	Nordfyns	4.05	6.03	6.32	7.06	6.73	7.25	5.99	5.60	5.80
F	Nyborg	4.45	6.94	6.31	10.45	7.95	9.08	10.81	10.05	9.89
F	Odense	5.76	8.22	5.31	7.20	6.03	8.72	8.15	5.06	5.52
F	Svendborg	4.24	4.74	5.68	7.78	8.25	9.69	9.31	8.31	8.49
C.C	Dragoer	2.45	3.09	3.88	4.91	4.63	4.72	4.93	5.74	6.69
C.C	Frederiksberg	2.12	3.08	2.64	2.94	2.66	3.15	3.01	4.17	6.23
C.C	Koebenhavn	1.94	1.58	2.01	2.02	2.23	2.00	1.80	3.75	3.14
C.C	Taarnby	2.10	2.78	3.27	3.78	3.36	4.25	4.34	5.10	5.52
C.S	Albertslund	1.86	2.36	2.35	3.76	3.07	3.35	4.13	4.37	4.29
C.S	Ballerup	2.27	2.13	2.52	3.29	5.62	6.94	8.25	7.90	8.13
C.S	Broendby	3.37	4.73	5.34	5.93	5.01	6.61	7.65	6.40	6.13
C.S	Gentofte	1.96	2.37	1.79	3.15	4.81	5.74	7.05	7.17	7.43
C.S	Gladsaxe	1.90	2.03	1.80	3.01	4.86	5.61	6.97	7.62	6.56
C.S	Glostrup	2.50	3.12	2.91	4.67	3.89	4.43	5.52	5.85	5.13
C.S	Herlev	3.02	2.91	2.71	4.13	5.30	7.19	10.44	8.38	8.65
C.S	Hoeje-Taastrup	2.07	2.96	3.50	4.35	3.53	5.77	4.04	5.35	4.68
C.S	Hvidovre	2.30	3.45	4.22	4.93	4.56	4.26	4.69	5.40	4.86
C.S	Ishoej	2.15	2.70	2.39	4.01	3.01	3.74	4.18	6.26	5.12
C.S	Lyngby-Taarbaek	2.28	3.02	2.13	3.58	6.52	6.14	7.84	8.18	8.25
C.S	Roedovre	2.77	2.81	2.58	3.48	4.77	5.28	10.67	7.89	7.07
C.S	Vallensbaek	2.44	2.27	2.73	3.81	3.36	4.59	4.58	5.61	5.03
N-J	Broenderslev	2.04	1.92	1.87	2.25	2.79	2.19	2.15	2.18	2.60
N-J	Frederikshavn	2.82	2.77	2.75	3.11	3.24	3.06	3.22	3.15	3.11
N-J	Hjoerring	2.09	2.41	2.27	2.57	2.55	2.92	2.75	2.99	2.70
N-J	Jammerbugt	2.58	2.62	3.81	3.12	3.09	2.45	2.45	2.51	2.24
N-J	Laesoe	3.32	3.33	3.08	5.31	7.21	3.91	3.98	3.83	3.79
N-J	Mariagerfjord	2.34	3.12	2.59	3.23	3.67	3.47	2.70	2.77	2.61
N-J	Morsoe	2.37	2.34	2.89	3.50	4.42	3.43	3.01	2.65	4.60
N-J	Rebild	1.98	2.61	2.57	3.22	2.84	2.84	2.43	2.23	2.29
N-J	Thisted	1.97	2.03	2.64	3.46	3.08	3.23	3.08	2.86	2.95
N-J	Vesthimmerlands	1.80	2.41	2.20	3.16	2.60	2.85	2.19	2.36	2.74
N-J	Aalborg	2.41	2.25	2.70	2.57	2.33	2.11	2.57	2.19	2.07
N-Z	Alleroed	3.70	3.53	2.54	4.77	5.14	5.03	4.82	6.19	6.74
N-Z	Egedal	2.05	2.01	1.82	2.28	4.02	5.15	7.15	6.62	7.44
N-Z	Fredensborg	3.85	4.00	3.19	5.40	4.63	4.89	6.07	7.23	6.76
N-Z	Frederikssund	4.75	4.49	3.77	6.41	6.83	6.77	7.89	8.44	8.43
N-Z	Furesoe	2.67	2.08	1.93	3.79	4.95	5.67	8.20	8.34	9.32
N-Z	Gribskov	5.02	4.77	3.86	6.38	6.60	7.20	9.99	9.84	9.78
N-Z	Halsnaes	6.22	6.21	4.82	6.32	8.28	9.41	9.46	8.91	11.66
N-Z	Helsingoer	5.71	5.17	3.60	5.69	6.48	8.28	8.92	8.85	9.17
N-Z	Hilleroed	3.74	3.78	2.56	4.20	3.78	4.16	5.36	5.48	5.11
N-Z	Hoersholm	5.33	5.41	3.83	5.47	9.04	6.25	7.09	8.03	8.81
N-Z	Rudersdal	2.92	3.30	2.18	5.29	4.96	6.41	7.20	7.57	8.52
E-J	Favrskov	2.62	2.93	3.06	4.80	5.68	3.20	3.10	2.97	2.96

Province	Municipality	2014	2015	2016	2017	2018	2019	2020	2021	2022
E-J	Hedensted	2.19	2.76	3.19	4.79	4.51	3.52	3.08	3.77	3.54
E-J	Horsens	1.73	2.79	2.72	4.68	3.79	2.58	2.09	3.41	3.47
E-J	Norddjurs	3.82	4.32	3.51	7.59	5.76	4.51	3.61	3.99	4.17
E-J	Odder	1.80	2.43	2.99	4.15	3.31	2.94	2.16	2.75	2.71
E-J	Randers	4.70	4.80	5.60	8.92	8.23	5.57	8.22	4.61	5.60
E-J	Samsoe	3.70	3.77	5.97	7.18	7.22	5.44	5.46	7.13	6.53
E-J	Silkeborg	2.51	3.92	4.34	4.28	4.28	4.34	4.14	3.37	3.58
E-J	Skanderborg	1.44	1.98	2.03	3.36	2.74	2.14	1.70	2.13	2.20
E-J	Syddjurs	2.52	2.48	3.01	4.99	4.47	3.85	2.78	3.40	3.52
E-J	Aarhus	1.40	2.09	2.28	3.38	2.81	2.31	1.52	1.36	1.45
E-Z	Greve	2.85	3.39	3.19	4.77	4.39	4.49	3.10	4.98	6.97
E-Z	Koege	2.39	3.20	2.71	4.08	4.94	4.83	3.89	6.72	7.71
E-Z	Lejre	2.62	3.15	2.97	3.93	4.94	4.04	2.87	3.02	3.44
E-Z	Roskilde	3.37	4.59	4.46	6.16	6.76	4.08	5.11	3.77	5.49
E-Z	Solroed	1.97	2.12	2.34	2.85	3.76	2.75	2.74	4.49	4.68
S-J	Billund	1.86	1.92	2.47	3.60	3.76	4.05	5.22	5.58	6.56
S-J	Esbjerg	1.72	1.95	2.28	3.02	3.89	3.24	5.56	5.67	6.46
S-J	Fanoe	2.80	2.93	6.23	4.62	7.23	5.59	8.00	6.35	6.35
S-J	Fredericia	5.06	5.22	5.58	8.26	7.45	8.44	8.10	8.26	8.49
S-J	Haderslev	2.74	2.78	3.52	4.77	5.88	5.35	4.15	3.18	3.76
S-J	Kolding	5.21	5.45	6.86	8.46	9.08	8.68	8.99	8.46	7.49
S-J	Soenderborg	3.23	3.48	3.16	4.71	5.72	5.95	4.20	3.79	3.08
S-J	Toender	2.86	2.43	2.93	3.89	3.78	4.30	3.77	2.73	3.82
S-J	Varde	1.70	1.75	2.07	2.71	3.40	3.35	4.86	4.79	5.11
S-J	Vejen	3.15	4.03	4.39	5.47	6.41	6.90	6.10	7.06	6.37
S-J	Vejle	2.37	4.25	3.67	6.04	5.10	5.04	8.17	5.30	4.57
S-J	Aabenraa	2.60	2.77	3.20	4.14	4.87	4.24	3.20	4.01	2.85
WS-Z	Faxe	2.05	2.01	2.59	4.15	3.77	3.67	3.21	5.21	5.17
WS-Z	Guldborgsund	2.74	2.66	3.99	5.56	4.59	3.81	3.35	3.53	4.79
WS-Z	Holbaek	2.67	2.63	2.86	3.69	4.17	3.45	3.30	3.40	4.03
WS-Z	Kalundborg	4.40	4.74	4.34	6.73	6.92	4.97	4.13	6.43	7.49
WS-Z	Lolland	3.64	4.32	5.18	5.58	7.21	6.00	3.88	4.62	6.14
WS-Z	Naestved	2.08	3.01	3.42	4.01	4.19	3.56	3.46	4.53	4.87
WS-Z	Odsherred	3.46	3.80	3.77	5.80	6.09	4.39	4.35	4.67	5.31
WS-Z	Ringsted	2.77	3.00	2.66	4.24	4.18	3.35	2.74	5.59	4.53
WS-Z	Slagelse	2.34	2.77	2.49	3.21	3.46	3.43	2.59	5.00	4.26
WS-Z	Soroe	2.86	3.72	3.17	4.17	4.19	3.87	3.39	4.14	3.97
WS-Z	Stevns	2.03	2.76	3.35	4.42	4.96	4.28	3.17	5.08	6.23
WS-Z	Vordingborg	2.34	3.28	2.94	5.59	6.01	4.80	3.26	3.59	5.34
W-J	Herning	2.27	2.78	3.49	2.33	3.06	2.55	3.49	4.05	3.85
W-J	Holstebro	2.11	2.66	2.84	2.64	2.36	2.25	3.27	3.78	3.40
W-J	Ikast-Brande	1.95	3.87	3.64	3.94	3.89	3.31	4.43	5.19	4.90
W-J	Lemvig	2.32	3.68	3.86	4.40	3.76	4.34	4.60	6.88	6.61
W-J	Ringkoebing-Skjern	2.04	3.00	2.44	3.34	3.65	3.15	3.63	5.15	4.64
W-J	Skive	2.87	3.97	4.29	5.59	5.57	5.62	3.35	3.23	5.38
W-J	Struer	2.03	3.79	3.21	3.18	3.01	2.58	3.41	4.25	4.21
W-J	Viborg	1.73	2.29	3.83	3.72	3.24	2.57	2.17	2.11	3.26

Provinces: **B** Bornholm, **F** Funen, **C.C** Copenhagen City, **C.S** Copenhagen 165

Surroundings, **N-J** North Jutland, **N-Z** North Zealand, **E-J** East Jutland, **E-Z** East Zealand, **S-J** South Jutland, **WS-Z** West and South Zealand, **W-J** West Jutland. **S-J** South Jutland, **WS-Z** West and South Zealand, **W-J** West Jutland.

S23 Testing-unadjusted case reporting, look-up table.

Table 9. Case reporting (estimated): reported cases/100 000 inhabitants.

Province	Municipality	2014	2015	2016	2017	2018	2019	2020	2021	2022
B	Bornholm	2.03	2.45	2.50	3.49	3.50	3.50	3.27	3.60	3.92
B	Christiansoe	1.94	2.14	2.34	3.30	3.12	3.00	3.33	3.30	3.33
F	Aeroe	4.39	4.88	4.95	7.19	7.93	7.23	7.33	7.45	8.14
F	Assens	4.61	5.00	5.08	7.41	6.90	8.65	10.19	10.37	8.98
F	Faaborg-Midtfyn	4.69	4.70	5.15	7.34	6.36	8.45	7.52	8.68	9.90
F	Kerteminde	4.55	7.07	6.07	9.86	7.18	8.55	8.08	8.20	7.54
F	Langeland	5.24	5.77	7.04	8.23	9.86	9.03	11.04	10.34	8.67
F	Middelfart	6.72	6.20	4.94	9.07	9.84	9.85	7.94	8.75	7.51
F	Nordfyns	4.36	5.64	7.35	7.84	8.56	8.59	7.50	7.07	7.66
F	Nyborg	5.30	7.95	6.84	11.49	8.00	8.01	10.27	9.63	9.54
F	Odense	5.90	8.47	4.86	6.86	6.05	9.20	8.93	5.29	6.86
F	Svendborg	4.86	4.86	5.73	8.56	8.58	9.18	8.22	7.21	7.66
C.C	Dragoer	2.17	2.37	2.64	3.41	3.45	3.79	3.48	3.87	3.52
C.C	Frederiksberg	1.90	2.60	2.23	2.97	2.78	3.01	3.29	3.10	5.38
C.C	Koebenhavn	2.06	1.36	1.90	1.88	2.49	1.92	1.67	3.89	3.12
C.C	Taarnby	2.18	2.35	2.36	3.26	2.99	3.85	3.02	3.61	3.58
C.S	Albertslund	2.34	2.57	2.88	4.07	3.77	4.14	4.62	3.97	3.95
C.S	Ballerup	3.57	3.31	3.38	5.01	7.35	7.34	5.95	5.92	6.37
C.S	Broendby	3.53	4.16	3.85	4.42	4.44	5.71	6.32	4.94	5.27
C.S	Gentofte	2.84	2.82	2.85	4.78	5.16	5.60	5.71	6.19	6.65
C.S	Gladsaxe	2.66	3.10	2.87	4.46	6.71	5.49	4.84	6.02	5.58
C.S	Glostrup	3.25	3.52	3.55	4.53	4.94	4.95	4.96	4.94	4.88
C.S	Herlev	3.64	3.59	3.62	7.05	5.47	5.92	8.28	5.99	6.51
C.S	Hoeje-Taastrup	2.64	3.40	3.44	3.98	3.68	5.51	3.76	4.43	4.35
C.S	Hvidovre	2.37	3.03	3.05	3.82	4.47	3.78	3.83	4.89	4.17
C.S	Ishoej	2.58	2.81	2.59	4.00	3.68	3.71	3.81	5.51	3.88
C.S	Lyngby-Taarbaek	3.23	3.76	3.45	5.41	7.72	5.76	6.25	7.18	7.53
C.S	Roedovre	3.34	3.60	3.31	5.31	4.44	4.75	9.53	6.06	5.12
C.S	Vallensbaek	3.17	2.88	2.95	4.56	4.12	4.10	4.18	5.10	4.26
N-J	Broenderslev	1.95	1.77	1.97	2.53	2.79	2.55	2.60	2.86	2.62
N-J	Frederikshavn	2.54	2.55	2.80	3.29	3.90	3.34	3.71	3.78	3.79
N-J	Hjoerring	1.67	2.18	2.22	2.57	2.84	3.37	3.17	3.79	3.25
N-J	Jammerbugt	1.86	1.86	1.89	2.92	3.21	2.96	3.03	3.37	2.83
N-J	Laesoe	3.10	3.33	3.39	5.45	5.10	5.11	5.26	5.37	5.39
N-J	Mariagerfjord	2.46	2.70	2.30	3.23	4.22	3.59	3.69	4.10	3.78
N-J	Morsoe	2.37	2.37	2.42	3.44	3.81	3.50	4.28	3.63	4.34
N-J	Rebild	1.82	2.00	2.23	3.15	2.86	4.07	3.16	2.91	3.20
N-J	Thisted	1.67	1.83	2.23	3.40	2.89	3.17	2.97	2.76	3.01
N-J	Vesthimmerlands	1.74	1.91	1.95	3.30	2.78	3.07	2.90	3.54	2.96

Province	Municipality	2014	2015	2016	2017	2018	2019	2020	2021	2022
N-J	Aalborg	1.75	1.61	2.45	2.90	2.72	2.24	3.08	2.57	2.40
N-Z	Alleroed	3.14	3.44	3.51	6.97	5.90	5.39	5.08	5.59	6.64
N-Z	Egedal	3.20	3.21	3.02	4.19	5.01	5.45	5.58	5.20	6.54
N-Z	Fredensborg	3.36	3.70	3.77	6.56	5.63	5.23	5.81	5.89	6.37
N-Z	Frederikssund	4.22	3.93	4.34	6.87	6.44	6.00	6.67	7.86	6.80
N-Z	Furesoe	3.49	3.48	3.49	4.78	5.65	5.68	6.28	6.87	8.52
N-Z	Gribskov	4.04	4.08	4.15	7.79	6.27	6.85	10.15	8.94	8.98
N-Z	Halsnaes	4.46	5.78	4.98	6.29	7.49	8.82	9.03	7.25	11.60
N-Z	Helsingoer	4.88	4.53	3.95	7.03	7.10	9.87	9.46	7.38	7.94
N-Z	Hilleroed	3.21	3.80	3.25	4.81	4.46	4.48	5.33	4.97	4.53
N-Z	Hoersholm	4.22	5.05	4.70	6.49	9.84	6.56	6.18	6.83	6.85
N-Z	Rudersdal	3.51	4.46	3.84	7.43	5.20	6.91	5.64	6.54	7.00
E-J	Favrskov	2.25	2.69	2.30	4.84	5.69	3.52	3.59	3.35	3.63
E-J	Hedensted	2.83	2.81	2.89	5.51	4.37	4.76	4.12	4.17	3.83
E-J	Horsens	1.79	2.93	2.14	4.19	3.63	2.87	2.94	3.44	3.65
E-J	Norddjurs	3.21	4.16	3.26	7.90	4.92	4.99	5.14	6.11	4.81
E-J	Odder	1.85	2.04	2.08	3.56	2.98	3.28	3.07	3.14	3.14
E-J	Randers	4.23	4.52	4.87	8.25	7.01	6.25	8.92	4.98	5.29
E-J	Samsoe	2.62	2.86	3.25	4.28	4.31	4.31	4.43	4.42	4.39
E-J	Silkeborg	2.67	2.68	2.72	3.64	3.65	3.92	3.69	3.93	3.65
E-J	Skanderborg	1.57	1.88	2.09	3.39	2.65	2.43	2.47	2.49	2.70
E-J	Syddjurs	2.54	2.56	2.85	5.10	4.00	4.35	3.76	3.81	4.13
E-J	Aarhus	1.34	1.96	2.33	3.26	2.66	2.50	1.79	1.58	1.78
E-Z	Greve	2.99	4.19	3.03	5.71	4.95	5.37	4.31	5.52	5.95
E-Z	Koege	2.99	3.52	2.79	3.82	5.27	5.29	5.40	6.28	6.28
E-Z	Lejre	2.92	2.93	2.99	4.61	5.05	5.09	4.34	4.78	4.34
E-Z	Roskilde	3.10	3.63	4.25	6.26	6.29	4.86	7.24	4.62	6.41
E-Z	Solroed	2.89	3.46	2.91	4.13	4.96	4.13	4.20	4.63	4.23
S-J	Billund	2.37	2.62	2.91	3.74	4.52	3.77	4.24	5.12	4.25
S-J	Esbjerg	2.14	2.70	2.74	3.88	4.75	3.39	5.17	3.51	4.90
S-J	Fanoe	4.08	4.58	4.68	6.72	7.47	6.84	8.54	7.17	7.28
S-J	Fredericia	5.73	5.31	5.38	7.16	7.72	7.75	7.36	7.96	8.55
S-J	Haderslev	2.76	2.77	3.34	5.31	5.77	5.39	4.01	4.43	5.59
S-J	Kolding	5.03	4.37	6.13	8.15	8.65	7.75	8.30	7.91	6.63
S-J	Soenderborg	2.53	2.99	2.82	4.77	4.80	5.18	4.59	5.77	4.03
S-J	Toender	2.19	2.42	2.69	3.74	3.46	3.48	4.22	3.63	4.70
S-J	Varde	2.11	2.30	2.35	3.54	3.89	3.60	4.70	4.08	3.75
S-J	Vejen	2.77	3.31	4.34	5.04	5.95	6.42	4.40	5.66	4.82
S-J	Vejle	2.50	4.72	3.39	6.31	5.28	4.97	7.11	5.03	5.28
S-J	Aabenraa	2.40	2.62	2.68	3.98	3.99	4.34	3.79	6.09	3.57
WS-Z	Faxe	2.64	2.64	2.94	4.47	3.79	3.80	4.61	5.08	4.29
WS-Z	Guldborgsund	2.76	2.55	3.31	4.81	3.82	3.84	4.59	3.67	5.01
WS-Z	Holbaek	2.56	2.58	2.40	3.54	3.86	4.50	4.26	4.99	4.28
WS-Z	Kalundborg	3.41	4.03	3.47	6.98	5.60	4.83	4.95	7.23	7.34
WS-Z	Lolland	3.37	4.72	5.21	5.58	6.58	6.61	6.76	6.35	6.87
WS-Z	Naestved	2.34	2.97	3.02	3.75	4.05	4.37	4.46	4.20	4.83
WS-Z	Odsherred	2.73	3.00	3.34	5.03	5.09	4.33	4.84	5.81	4.95
WS-Z	Ringsted	2.64	3.17	2.69	4.10	4.48	4.07	4.58	5.98	4.26
WS-Z	Slagelse	2.36	2.35	2.20	3.23	3.24	3.79	3.31	4.86	4.19
WS-Z	Soroe	2.98	3.25	2.76	4.25	4.28	3.93	4.79	4.83	4.08
WS-Z	Stevns	2.60	3.14	2.91	4.50	4.52	5.00	4.29	4.33	4.71

Province	Municipality	2014	2015	2016	2017	2018	2019	2020	2021	2022
WS-Z	Vordingborg	2.53	3.01	2.81	5.80	4.99	4.66	5.19	4.49	4.88
W-J	Herning	2.40	1.88	2.45	2.60	3.31	3.33	3.94	3.19	3.17
W-J	Holstebro	1.97	1.81	2.01	3.02	2.79	2.57	3.37	3.13	2.64
W-J	Ikast-Brande	2.27	3.55	2.78	3.87	4.23	4.24	5.11	4.03	4.00
W-J	Lemvig	2.31	2.33	2.89	3.43	4.17	3.51	3.97	3.69	4.46
W-J	Ringkoebing-Skjern	2.39	2.20	2.44	3.96	3.68	4.00	3.49	4.17	3.55
W-J	Skive	2.43	3.17	2.70	4.83	3.81	5.76	3.95	3.70	4.40
W-J	Struer	1.56	1.72	1.76	2.53	2.56	2.61	2.69	2.76	2.77
W-J	Viborg	1.68	1.83	2.19	3.40	2.97	2.76	3.05	3.10	3.60

Provinces: **B** Bornholm, **F** Funen, **C.C** Copenhagen City, **C.S** Copenhagen Surroundings, **N-J** North Jutland, **N-Z** North Zealand, **E-J** East Jutland, **E-Z** East Zealand, **S-J** South Jutland, **WS-Z** West and South Zealand, **W-J** West Jutland. **S-J** SouthJutland, **WS-Z** West and South Zealand, **W-J** West Jutland.

S24 Difference testing-unadjusted minus testing-adjusted case reporting, look-up table.

Table 10. Difference testing-unadjusted minus testing-adjusted case reporting (estimated): difference of reported cases/100 000 inhabitants.

Municipality	2014	2015	2016	2017	2018	2019	2020	2021	2022
Bornholm	0.10	-0.28	-1.48	-0.43	0.48	-0.30	-0.58	-1.02	-1.37
Christiansoe	-0.24	-0.11	0.08	0.52	0.67	-5.34	0.69	0.81	0.85
Aeroe	0.51	-0.09	-0.44	0.85	1.16	1.09	0.03	-0.11	0.34
Assens	0.58	0.12	-0.02	0.83	0.19	0.72	0.23	0.60	-0.36
Faaborg-Midtfyn	0.91	0.45	0.27	0.88	0.79	0.74	-0.61	0.12	0.70
Kerteminde	0.79	0.67	0.59	2.02	0.20	1.52	-0.81	-0.45	1.98
Langeland	0.59	-2.02	-1.98	-3.06	-1.83	-1.76	-0.91	-0.79	-1.29
Middelfart	0.95	0.25	-1.20	0.06	0.88	-0.32	-0.10	-0.31	-0.66
Nordfyns	0.30	-0.39	1.03	0.78	1.82	1.33	1.51	1.47	1.86
Nyborg	0.85	1.00	0.53	1.04	0.05	-1.07	-0.54	-0.42	-0.35
Odense	0.14	0.25	-0.45	-0.34	0.01	0.49	0.78	0.23	1.34
Svendborg	0.62	0.12	0.04	0.78	0.33	-0.51	-1.08	-1.10	-0.83
Dragoer	-0.28	-0.72	-1.24	-1.50	-1.18	-0.93	-1.45	-1.87	-3.17
Frederiksberg	-0.22	-0.48	-0.41	0.03	0.12	-0.14	0.28	-1.06	-0.85
Koebenhavn	0.12	-0.22	-0.10	-0.14	0.26	-0.08	-0.13	0.14	-0.02
Taarnby	0.08	-0.43	-0.91	-0.52	-0.38	-0.40	-1.32	-1.48	-1.94
Albertslund	0.48	0.21	0.54	0.32	0.70	0.79	0.48	-0.40	-0.34
Ballerup	1.31	1.18	0.86	1.72	1.73	0.40	-2.30	-1.98	-1.76
Broendby	0.16	-0.58	-1.49	-1.51	-0.57	-0.90	-1.33	-1.46	-0.86
Gentofte	0.88	0.45	1.07	1.63	0.35	-0.14	-1.34	-0.98	-0.77
Gladsaxe	0.76	1.07	1.07	1.45	1.86	-0.12	-2.13	-1.60	-0.98
Glostrup	0.75	0.40	0.64	-0.14	1.05	0.52	-0.56	-0.91	-0.24
Herlev	0.63	0.68	0.91	2.92	0.17	-1.27	-2.16	-2.39	-2.15
Hoeje-Taastrup	0.57	0.44	-0.06	-0.37	0.15	-0.26	-0.28	-0.91	-0.33

Municipality	2014	2015	2016	2017	2018	2019	2020	2021	2022
Hvidovre	0.07	-0.42	-1.17	-1.11	-0.09	-0.48	-0.85	-0.52	-0.70
Ishoej	0.43	0.11	0.19	-0.01	0.67	-0.03	-0.37	-0.75	-1.24
Lyngby-Taarbaek	0.95	0.74	1.32	1.82	1.21	-0.38	-1.59	-1.00	-0.72
Roedovre	0.57	0.79	0.74	1.83	-0.33	-0.53	-1.14	-1.83	-1.95
Vallensbaek	0.73	0.61	0.22	0.74	0.76	-0.49	-0.40	-0.52	-0.77
Broenderslev	-0.09	-0.15	0.10	0.28	0.00	0.36	0.45	0.69	0.02
Frederikshavn	-0.28	-0.23	0.06	0.18	0.66	0.28	0.49	0.63	0.68
Hjoerring	-0.42	-0.23	-0.06	0.00	0.29	0.45	0.43	0.80	0.55
Jammerbugt	-0.72	-0.76	-1.92	-0.20	0.12	0.52	0.58	0.86	0.59
Laesoe	-0.22	0.00	0.31	0.14	-2.10	1.20	1.28	1.54	1.60
Mariagerfjord	0.12	-0.42	-0.28	0.00	0.55	0.13	0.99	1.33	1.17
Morsoe	0.00	0.02	-0.47	-0.06	-0.60	0.07	1.27	0.99	-0.25
Rebild	-0.16	-0.60	-0.34	-0.07	0.02	1.22	0.73	0.68	0.91
Thisted	-0.30	-0.20	-0.41	-0.06	-0.19	-0.06	-0.11	-0.09	0.06
Vesthimmerlands	-0.06	-0.50	-0.25	0.14	0.18	0.22	0.72	1.17	0.23
Aalborg	-0.66	-0.64	-0.25	0.34	0.40	0.13	0.52	0.38	0.33
Alleroed	-0.56	-0.09	0.98	2.20	0.77	0.36	0.25	-0.60	-0.10
Egedal	1.15	1.20	1.20	1.90	0.99	0.30	-1.56	-1.42	-0.90
Fredensborg	-0.49	-0.30	0.58	1.16	1.00	0.34	-0.26	-1.34	-0.40
Frederikssund	-0.53	-0.56	0.57	0.46	-0.39	-0.77	-1.23	-0.58	-1.64
Furesoe	0.82	1.40	1.56	0.99	0.70	0.01	-1.92	-1.47	-0.79
Gribskov	-0.98	-0.69	0.29	1.41	-0.33	-0.35	0.16	-0.91	-0.80
Halsnaes	-1.77	-0.43	0.15	-0.02	-0.79	-0.59	-0.43	-1.66	-0.06
Helsingoer	-0.83	-0.64	0.35	1.34	0.62	1.59	0.54	-1.47	-1.23
Hilleroed	-0.53	0.01	0.69	0.62	0.69	0.32	-0.03	-0.51	-0.58
Hoersholm	-1.11	-0.36	0.87	1.02	0.80	0.31	-0.91	-1.20	-1.96
Rudersdal	0.59	1.17	1.66	2.15	0.24	0.50	-1.56	-1.02	-1.52
Favrskov	-0.37	-0.24	-0.76	0.04	0.01	0.32	0.50	0.38	0.67
Hedensted	0.65	0.05	-0.30	0.73	-0.13	1.24	1.04	0.40	0.29
Horsens	0.06	0.14	-0.58	-0.49	-0.16	0.29	0.85	0.04	0.18
Norddjurs	-0.61	-0.16	-0.26	0.31	-0.84	0.48	1.53	2.12	0.64
Odder	0.05	-0.39	-0.92	-0.59	-0.33	0.34	0.92	0.39	0.43
Randers	-0.47	-0.27	-0.72	-0.66	-1.22	0.69	0.70	0.37	-0.31
Samsoe	-1.08	-0.91	-2.73	-2.90	-2.91	-1.13	-1.03	-2.72	-2.14
Silkeborg	0.16	-1.24	-1.62	-0.64	-0.63	-0.42	-0.45	0.56	0.06
Skanderborg	0.13	-0.10	0.06	0.04	-0.09	0.30	0.77	0.36	0.50
Syddjurs	0.02	0.08	-0.16	0.11	-0.47	0.50	0.99	0.41	0.62
Aarhus	-0.06	-0.14	0.05	-0.12	-0.15	0.19	0.26	0.23	0.33
Greve	0.15	0.80	-0.16	0.94	0.55	0.88	1.21	0.55	-1.01
Koege	0.59	0.31	0.07	-0.26	0.32	0.46	1.50	-0.43	-1.43
Lejre	0.30	-0.22	0.02	0.68	0.12	1.05	1.47	1.76	0.90
Roskilde	-0.27	-0.96	-0.22	0.10	-0.47	0.78	2.13	0.85	0.92
Solroed	0.92	1.33	0.57	1.28	1.20	1.38	1.46	0.14	-0.45
Billund	0.52	0.70	0.44	0.14	0.76	-0.27	-0.98	-0.46	-2.32
Esbjerg	0.42	0.75	0.47	0.86	0.86	0.15	-0.39	-2.16	-1.56
Fanoe	1.29	1.65	-1.55	2.10	0.23	1.25	0.54	0.82	0.93
Fredericia	0.68	0.09	-0.19	-1.10	0.28	-0.69	-0.75	-0.30	0.05
Haderslev	0.03	-0.01	-0.18	0.54	-0.11	0.04	-0.14	1.25	1.84
Kolding	-0.19	-1.08	-0.74	-0.31	-0.43	-0.93	-0.69	-0.55	-0.86
Soenderborg	-0.69	-0.48	-0.34	0.06	-0.92	-0.77	0.39	1.98	0.95

Municipality	2014	2015	2016	2017	2018	2019	2020	2021	2022
Toender	-0.68	-0.01	-0.25	-0.15	-0.31	-0.82	0.45	0.90	0.89
Varde	0.41	0.55	0.28	0.83	0.50	0.24	-0.16	-0.71	-1.36
Vejen	-0.37	-0.72	-0.05	-0.43	-0.46	-0.48	-1.70	-1.40	-1.55
Vejle	0.13	0.47	-0.29	0.27	0.18	-0.07	-1.07	-0.27	0.70
Aabenraa	-0.21	-0.16	-0.52	-0.16	-0.88	0.11	0.59	2.09	0.72
Faxe	0.59	0.63	0.35	0.33	0.02	0.13	1.40	-0.13	-0.88
Guldborgsund	0.02	-0.11	-0.68	-0.75	-0.76	0.04	1.24	0.13	0.22
Holbaek	-0.11	-0.05	-0.46	-0.15	-0.32	1.06	0.96	1.58	0.25
Kalundborg	-0.99	-0.71	-0.87	0.26	-1.31	-0.14	0.82	0.80	-0.15
Lolland	-0.27	0.40	0.03	0.00	-0.64	0.62	2.88	1.74	0.73
Naestved	0.26	-0.04	-0.40	-0.26	-0.14	0.81	1.00	-0.33	-0.04
Odsherred	-0.73	-0.80	-0.43	-0.76	-0.99	-0.06	0.49	1.14	-0.36
Ringsted	-0.13	0.16	0.04	-0.14	0.30	0.72	1.84	0.40	-0.27
Slagelse	0.02	-0.42	-0.30	0.02	-0.21	0.36	0.72	-0.14	-0.08
Soroe	0.12	-0.46	-0.41	0.08	0.09	0.06	1.40	0.69	0.11
Stevns	0.58	0.37	-0.45	0.08	-0.43	0.72	1.12	-0.75	-1.51
Vordingborg	0.18	-0.26	-0.13	0.21	-1.02	-0.14	1.93	0.90	-0.46
Herning	0.13	-0.90	-1.04	0.27	0.25	0.78	0.46	-0.86	-0.68
Holstebro	-0.14	-0.85	-0.83	0.38	0.43	0.32	0.10	-0.65	-0.76
Ikast-Brande	0.32	-0.32	-0.86	-0.07	0.34	0.93	0.67	-1.16	-0.89
Lemvig	-0.01	-1.34	-0.97	-0.97	0.42	-0.83	-0.64	-3.19	-2.15
Ringkoebing-Skjern	0.35	-0.81	0.00	0.62	0.03	0.84	-0.13	-0.98	-1.09
Skive	-0.44	-0.80	-1.59	-0.75	-1.76	0.14	0.60	0.48	-0.97
Struer	-0.47	-2.07	-1.46	-0.65	-0.45	0.03	-0.72	-1.50	-1.45
Viborg	-0.05	-0.46	-1.64	-0.32	-0.26	0.19	0.89	0.99	0.34
