# Supplementary material for: Diagnostic testing intensity for Legionnaires’ disease: Spatio-temporal assessment and its effect on surveillance case reporting, Denmark, 2014–2022
Source: PLoS One. 2026 Apr 17;21(4):e0346580. doi: 10.1371/journal.pone.0346580 (PMC13089883; doi:10.1371/journal.pone.0346580)
Supplement: Attachment — Submitted filename: Response to Reviewers.docx [file pone.0346580.s002.docx]

Dear Academic Editor,

We would like to thank you and the Reviewer for the valuable comments.

We submit a revised version of our manuscript, taking into account the points raised during the review process. We have carefully addressed the comments and reply below to each of them, clarifying also what was adapted in the manuscript.

**Comments by Academic Editor:**

1. **Please ensure that your manuscript meets PLOS ONE's style requirements, including those for file naming.**

We have edited the manuscript according to PLOS ONE’s style requirements. Files were renamed as required. We numbered the tables and figures and uploaded them separately. Textual changes are reflected in the revision file labelled 'Revised Manuscript with Track Changes'.

**2. We note that Figures 2, 4, 5, 6, and 7 in your submission contain map images which may be copyrighted. All PLOS content is published under the Creative Commons Attribution License (CC BY 4.0), which means that the manuscript, images, and Supporting Information files will be freely available online, and any third party is permitted to access, download, copy, distribute, and use these materials in any way, even commercially, with proper attribution. For these reasons, we cannot publish previously copyrighted maps or satellite images created using proprietary data.**

We have replaced the basemap of the Danish municipalities with data adapted from OpenStreetMap, available under the Open Database License, and we have added this information in the map legend. Furthermore, we have inserted the following information in the Material and Methods section: *“*The basemap of the Danish municipalities was adapted from OpenStreetMap, available under the Open Database License (openstreetmap.org/copyright).”

**3. Please ensure that you refer to Figures 5, 6, and 7 in your text as, if accepted, production will need this reference to link the reader to the figures.**

We have inserted reference to Figure 5. We have replaced the reference to Figure 8 and 9 by Figure 6 and 7, respectively.

**4. Please upload a copy of Figures 8 and 9, to which you refer in your text on pages 19 and 20. If the figure is no longer to be included as part of the submission please remove all reference to it within the text.**

 We have replaced the reference to Figures 8 and 9 by Figure 6 and 7, respectively.

**5. Please remove all personal information, ensure that the data shared are in accordance with participant consent, and re-upload a fully anonymized data set.**

We have reviewed the data shared through the manuscript, the supplementary information, and the repositories, considering the Data Policy and the guidance in the recommended article. The data provided are anonymized.

We provide observed data counts in Table 1 and 2 in the manuscript and Tables 1-4 in the Supplementary Information S4-S6. Further, in the Supplementary Information S21-S24 we provide extensive look-up tables with the exact values of the estimates as shown in the maps of the manuscript and further results from sensitivity analyses. Demographic and map data, along with computing code, are available at the GitHub and Zenodo repository. For access to additional data, we explain in the data availability statement how these can be requested.

**6. If the reviewer comments include a recommendation to cite specific previously published works, please review and evaluate these publications to determine whether they are relevant and should be cited.**

The reviewer did not include a recommendation to cite specific previously published works.

**Comments by Reviewer #1**

**Robesyn et al. present an interesting paper considering heterogeneity in LD testing and case reporting in space/time in Denmark. The analysis is of interest and benefits from multiple national-level data sources. I have the below comments which I hope will strengthen the manuscript further:**

We are grateful for the comments and acknowledge that addressing them has significantly improved the manuscript.

***Abstract:***

**- Please could you briefly define (perhaps in brackets after ‘Legionella Test’) what legionella tests you are including (i.e. UAT and PCRs).**

Thank you for highlighting this important aspect. We have inserted the test types as follows: **“**persons receiving at least one *Legionella* urinary antigen or PCR test per 100,000 inhabitants.”

**- ‘Conform a positive time effect’ does not seem a grammatically correct ending to this sentence – please improve.**

We have replaced this by “reflecting an upward trend.”

***Introduction:***

**- Typo line 44: ‘unknown to WHAT extent’ not ‘which’ extent**

We have replaced the word, both in the Introduction and in the Abstract.

**- Reformulation proposal lines 45-48: ‘Despite the global occurrence [2], the clinical severity [3], the public health burden (mostly attributable to years of life lost [4, 5]), and the treatable and preventable character of this waterborne respiratory disease, many low- and middle-income countries have no effective surveillance in place.’**

We agree that the suggested reformulation improves the readability and have adopted the proposal.

**- Line 48: add ‘within’ after including**

We have inserted the word.

***Methods:***

**- Lines 137-138 ‘Latent Legionnaires’ disease incidence’. We would not typically refer to LD as being ‘latent’ as we would for other communicable diseases like TB. I think ‘undiagnosed’ may be more appropriate for the presumed meaning behind the sentence. Please amend here and elsewhere in the manuscript where LD is referred to as latent.**

Indeed, the meaning of ‘latent’ in our manuscript does not refer to the infection status of an individual. Instead, it refers to the unmeasured or hidden disease generating process or true disease incidence in the population, which is our surveillance target of interest that we aim to estimate. To avoid confusion, we have replaced the word ‘latent’ by ‘true’ Legionnaires disease incidence. Similarly, we have also adapted the formulation of latent state in the Discussion.

**- Have case definitions remained consistent throughout the study period? Are they amended in the context of LD outbreaks?**

The case definition of Legionnaires’ disease used in Denmark, including in the context of outbreaks, is based on the official EU surveillance case definition. Outbreak case definitions differ mainly by restriction in time, place and person, but not in their clinical and laboratory criteria. Throughout the study period there was one change in the EU case definition, namely in 2018. The 2002 definition, valid until 25/07/2018, defined both Legionnaires’ disease and Pontiac fever, but this has not affected our study data, which is focussed on Legionnaires’ disease only. In both versions, a positive urinary antigen test was considered a confirmatory case criterium, while a positive PCR test classified the case as probable. This distinction in case classification was not deemed relevant to our study and was therefore not used. We note here that a large proportion (around 30-40%) of the PCR-positive cases in Denmark are confirmed by culture.

We have inserted the 2002 definition as reference in the Introduction of our manuscript, for completeness. We added a clarification in the Methods section: “Cases fulfilled the EU case definition of confirmed or probable case. Throughout the study period, the EU case definition considered a positive urinary antigen test as a confirmed case criterium, and a positive PCR test as a probable case criterium, but this distinction in case classification was not deemed relevant to our study and was therefore not used.”

**- Lines 143-144 should be ‘both’ not ‘either’.**
Indeed, both may influence the reported case count. We have replaced the word.

***Results:***

**- Were there changes in the proportion of probable/confirmed cases over time?**

For this study, we did not collect the variable ‘case classification’ but we can provide relevant background. Per the case definition in use during the study period, the proportion of probable and confirmed cases is largely (but not only) defined by the test types used: a positive urinary antigen test or a culture with isolation of *Legionella* spp. contributes to the case classification ‘confirmed’, while a positive PCR test contributes to a case being labelled as ‘probable’. As the proportion of urinary antigen test decreased and the proportion of PCR increased over the study period, the proportion of ‘confirmed’ cases has decreased. However, the binary surveillance classification ‘confirmed/probable case’ does not capture sufficiently the complexity of the test sensitivity and specificity of the different tests for the variety of *Legionella* species and serogroups causing clinical Legionnaires’ disease. We describe the most salient changes in use of test types over time in the paragraph before table 1, and we refer for more detail to Table 3 in the Supplementary Information S6 where we report on the within-year test profiles of the tested persons.

**- Line 303-304 please can you explain the sentence 83% of all positive tested persons in the database. Does this mean that the reported number (2183) is only 83% of the number of cases identified via the MiBa? I thought in lines 167-169 you stated the difference between the number of cases from both sources were negligible?**

Thank you for the question. To explain the difference, we refer to three nodes in Figure 1: ‘Pos Leg-tested’ or people who have tested positive for a Legionella test, ‘Diagnosed LD’ or people who were found to have Legionnaires’ disease by their clinician, and ‘Reported LD’ or people who’s diagnosis was recorded in the surveillance database by the Department of Infection Epidemiology and Prevention, Statens Serum Institut.

There is a significant difference between the number of positive Legionella tested persons and diagnosed cases (for which the reasons are explained in the manuscript), but a negligible difference between the number of diagnosed cases and reported cases.

We understand that our statement about ‘automated electronic reporting of data from the national microbiological database’ was misleading and did not capture that there is still a verification of the diagnosis with the clinician. The automated ‘detection algorithm’ of positive tests on the national microbiological database does not lead to automated ‘case reporting’ in the surveillance system but to verification of the diagnosis with the clinician.

We have corrected our manuscript text: “However, this is assumed negligible in Denmark since 2014 due to an automated detection algorithm on the national microbiological database, whereby for each positive test, the diagnosis is verified with the clinician.”

**- “To aid understanding and readability, I think a more formal definition of RR*T* would be helpful. What is it relative to? i.e. what number would give an RRT of 1? I have the same point for RR*C*.”**

We agree that the link between the model equations in the Methods and the terminology in the Results section was not explicit enough. The value of one for the relative risk (RR) refers to testing intensity or case reporting as expected based on the intensity or reporting in the whole study area. We have now inserted in the Methods section a clarifying text, with more formal definitions:

“In the Results section, we call the relative risk from the two models, respectively, the ‘relative testing intensity (RR_T_)’ and the ‘relative case reporting (RR_C_)’, i.e. the multiplicative factor for a specific year and municipality as compared to the expected value. A value of two means that the testing intensity or case reporting was twice as large as expected from the overall intensity/reporting in the whole country.”

**- Figure 3(b) the graph appears to have a more natural transition point somewhere between 1100-1200 tested persons/100,000. What is the rationale behind identifying 1000 as the transition point? That appears to still be on the upward slant of the curve.**

We have been hesitant to make strong conclusions about the trend above a testing intensity above 1000 tested persons/100,000 taking into consideration the slightly lower point estimates beyond 1200 and the larger uncertainty interval (Fig 3, b and S14 Fig 17). We have edited the text to capture better the continued increase of the point estimate up to 1200:

“Further, we estimated that with increasing testing intensity, the relative case reporting (on log scale) increased (Fig 3, b and S14 Fig 17). The increasing relative case reporting is observed up to about 1000 to 1200 tested persons per 100 000 inhabitants, with relative case reporting of about 1.6 (log(RR_C_) = 0.5). At higher testing intensities, there is no further improvement in case reporting and there is larger uncertainty of the estimate.”

**I think this graph would benefit from being made significantly larger - my understanding is that this is one of the most significant findings in the paper.**

We agree that Fig 3, b is an essential finding and we have increased its size. At this occasion, we have also made the caption of Fig 3 more accurate.

**- Line 369 has ‘case case’**
Thank you for noting.

***Discussion:***

**- The continued change in UAT and PCR testing over time is very interesting and deserves more comment/explanation in the discussion.**

We fully agree and have elaborated on this point in the Discussion as follows:

“Whereas the population of Denmark between 2014 and 2022 has increased with 4 %, the number of persons tested for Legionella in a given year has increased with 141%. While diagnostic testing intensity may not be known, a similar pattern may take place in other countries. Many reports and studies signalling apparent increased Legionnaires’ disease incidence without considering the testing intensity will therefore not reliably reflect true Legionnaires’ disease incidence.

Where since the beginning of the century urinary antigen test availability has drastically changed the diagnostic landscape, we described during our study period a shift from urinary antigen test use to PCR use, further boosted by PCR lab capacity created during the COVID-19 pandemic. We considered in our study the total testing intensity and did not focus on the test characteristics of the urinary antigen and PCR tests, but these are also key to understand the yield of the tests and thus the resulting reporting incidence.”

**- When describing the diagnostic testing intensity I think there should be some acknowledgement that this will depend to a certain degree on the underlying ‘true’ incidence of disease over time (i.e. if there are more cases in the population, then the curve of testing intensity against case reporting may appear different). An acknowledgement that this may differ in different times, in addition to different places, would be helpful.**

Indeed, in line with our DAG in Fig 1, the true disease will affect the testing intensity over time. We have reinforced the importance of the time dimension, in parallel to the spatial dimension, in the following way. Where we wrote “Even in our suggested alternative DAG, with physician’s awareness as unmeasured confounder, we do not capture that reporting of cases may trigger increased testing, followed by even higher case reporting.”, we have added “Such short as well as long changes over time may happen simultaneous to changes in true disease incidence, e.g. due to weather and climatic conditions, clearly making the inference of true disease incidence a major challenge.”

**- Line 542 typo: areal**

We believe the spelling is correct, referring to an area. Our modelling was based on counts in (administrative) area’s. When data were to come from hospitals, these counts would need to be attributed to a recruitment area to know the denominator for incidence calculation and modelling. We edited the sentence slightly to improve the structure.

**Conclusion:
- The conclusion ‘no substantial increase in true LD has occurred during the nine-year study period’ is too strong given the described limitations in the discussion, and is discordant with how the findings are more cautiously presented throughout the rest of the manuscript. Suggest amending to ‘suggests that’ or ‘evidence in favour of’.**

Thank you for the observation. For consistency with the presentation of the findings we have adapted the text, both in the conclusion and abstract, to “Our study results suggest that no substantial increase in true Legionnaires’ disease has occurred over the nine-year study period”.
