# Supplementary material for: Diagnostic testing intensity for Legionnaires’ disease: Spatio-temporal assessment and its effect on surveillance case reporting, Denmark, 2014–2022
Source: PLoS One. 2026 Apr 17;21(4):e0346580. doi: 10.1371/journal.pone.0346580 (PMC13089883; doi:10.1371/journal.pone.0346580)
Supplement: Attachment — Submitted filename: Response to reviewers_2.docx [file pone.0346580.s003.docx]

Dear Academic Editor,

We thank the second reviewer for the highly relevant comments. We believe that addressing them has significantly improved our manuscript.

**Reviewer**

**The study provides timely and important results for developing surveillance approaches to LD. The analysis uses an extensive national dataset of LD cases and diagnostic testing in Denmark. The modelling approach is novel and relevant to the field, the manuscript is generally well presented, and the figures and tables are clear. My comments mainly concern the robustness of the conclusions.**

**Major comments**

1. **Throughout the manuscript, especially in the abstract and conclusions, the interpretation shifts from** **testing-adjusted reporting (as a proxy for underlying incidence) to statements about “true” LD incidence. Even with the cautious wording (“suggest”), this may be read as a claim about the underlying disease process rather than the surveillance process.** **The analysis does not account for all uncertainties in diagnostic/ascertainment (e.g., it includes only cases that enter the diagnostic pathway), and the manuscript notes that adjusting for testing intensity can introduce collider bias. A revision of the abstract and conclusions to focus on testing-adjusted reporting and to reflect these limitations will avoid misinterpretation.**

We thank the reviewer for the thoughtful comment. We agree that the testing-adjusted reporting, our analysis outcome, is an imperfect proxy for the underlying incidence. Aspects like cases not presenting to healthcare, measurement error in the tests, dynamic interactions, unmeasured confounders, etc. are not accounted for, and we cannot exclude partial (i.e. depending on the strength of association between collider and descendant) collider bias. To avoid misinterpretation or overconfidence, we have removed ‘true’ where we refer to our work and meant latent or underlying incidence and adapted the wording in the abstract, the main text (original lines 127, 139, 148, 483, 579) and the conclusions.

Nevertheless, we do not want to obfuscate that one estimand of our surveillance study is indeed the underlying disease incidence, which -through our proxy- we tried to estimate to the best of our ability. We therefore feel it is needed to cautiously conclude on the underlying incidence or disease process, without suggesting it is ‘true’. The reasoning is similar to the argument that a causal analysis should, despite limitations, conclude on causal effects and not limit its conclusions to merely describing associations (Hernan, 2018). However, we have carefully avoided to refer to the underlying incidence or process in the Results section, and we refer in that section only to ‘testing-adjusted reporting’.

Reference: Hernán MA. Am J Public Health. 2018 May;108(5):616-619. doi: 10.2105/AJPH.2018.304337. Epub 2018 Mar 22. URL: https://pubmed.ncbi.nlm.nih.gov/29565659/

We are grateful for pointing at the limitation of the non-diagnostic path and we have inserted this aspect at the start of our limitations section. We take the opportunity to elaborate here a little further.

The surveillance case definition of Legionnaires’ disease indeed only aims to capture in-healthcare-tested disease. A proportion of legionella-caused pneumonia may not present at clinical care. However, in a country with good access to healthcare, pneumonia cases (contrary to legionellosis) not presenting at primary or secondary care are expected to be infrequent.

Another non-diagnostic pathway would be legionella-caused pneumonia presenting at healthcare but not receiving a Legionnaires’ disease test. Our hierarchical model allows -in principle-, through partial pooling, to mutually learn between areas with higher testing and areas with lower testing. This learning is not without limitations and future (simulation) studies may assess whether post-stratification, e.g. simulating a situation where all areas have very high testing, would provide better incidence estimates. Such studies could include measurement error models where test characteristics like sensitivity and specificity against different *Legionella* strains are taken into account.

In countries with generalised under-ascertainment, i.e. low testing in all or most areas, learning through partial pooling would be poor, and the non-diagnostic pathway would be an even more important limitation.

2. **The manuscript interprets spatially structured and space–time interaction effects as municipality-level clustering and potentially local environmental conditions. However, travel-associated cases, assigned to the municipality of residence rather than the likely exposure location, are included. Some spatial patterns may therefore reflect non-local exposures, affecting how “local” risk patterns are interpreted. Clarifying the rationale for including travel-associated cases, or providing a sensitivity analysis excluding them, would make the conclusions more reliable.**

Our primary estimand was the effect of testing intensity on the total Legionnaires’ disease reporting, the main indicator in many surveillance reports, and for that reason travel-associated cases were included. However, inferring local environmental risk from total case incidence may indeed be biased by the inclusion of travel-associated cases. To test the sensitivity of our results to this inclusion, we have now also made estimates excluding travel-associated cases and we have added this sensitivity analysis to the supplementary material. The estimated case reporting and the exceedance probabilities for both the relative case reporting and the spatio-temporal interaction are shown in S20 Fig 25-27, and at the end of this document. The results, e.g. the detection of Odense as cluster in 2015, remain consistent with our main conclusions. We have added this information also to the Discussion section.

3. **A relevant potential confounder that is not clearly addressed is urbanicity. Urbanicity may relate to both i) testing intensity and ii) exposure opportunities (e.g., higher density of cooling towers in urban areas). This could bias the estimated testing–case relationship and the interpretation of testing-adjusted spatial patterns.**

We agree that it is important to assess the potential biasing effect of urbanization degree on our estimates. Doing so on https://dagitty.net/, we found that adjustment for urbanization degree is only needed if we make the additional assumption that urbanization is related to the probability of being diagnosed, given being tested positive. Note that in the below graphs the arrow between Legionella infections and pneumonia is removed for illustrative purpose as in Figure 3 of Supplement S1, to highlight the relevant causal paths.

In the first graph below, where urbanization is influencing the presence of Legionella determinants (e.g. density of cooling towers) as well as the testing intensity (e.g. higher in cities), the path is blocked on Legionella determinants, whether or not we adjust for Legionella testing intensity.

In the alternative graph below, where the area determines the degree of urbanization, and the latter influences the testing intensity, we must adjust for urbanization to measure the direct path from municipality to reported cases (a). However, that is not needed anymore once we adjust for testing intensity (Leg-tested as white circle) (b).

| (a) |  |
| --- | --- |
| (b) |  |

Finally, when we consider that urbanization degree not only relates to testing intensity but also to the probability of being diagnosed, given being tested positive, then indeed bias is possible and adjustment for urbanization degree is needed.

We believe this may be a realistic assumption. At the time of our study this data was not available. We have mentioned this relevant factor in the Discussion, for consideration in future studies.

**Minor comments**

4. **L116: The prevention of LD depends not only on diagnostic guidelines and surveillance, but also on effective risk management of pathogenic Legionella species in engineered water systems. Please revise to reflect this broader prevention context.**

Thank you for the remark. We agree and have inserted this critical preventive element to the text.

5. **L257: The manuscript describes the testing effect as linear, while the SI indicates the results come from a nonlinear RW1 smooth over binned testing intensity. Please clarify the model specification in the main text to avoid confusion.**

Thank you for flagging this. We have corrected the notation and accompanying text.

6. **Figure 3 presents results on the logarithmic scale. For most readers, would it be easier to interpret on the arithmetic scale?**

Indeed, this will help the interpretation of our results. We have now plotted the relative testing intensity and the relative case reporting on the arithmetic scale.

**Three Figures added to supplement S20, sensitivity analyses:** relative case reporting and exceedance probabilities from data excluding travel-associated cases
